# Tyrosine kinase inhibition increases functional parkin-Beclin-1 interaction and enhances amyloid clearance and cognitive performance

**DOI:** 10.1002/emmm.201302771

**Published:** 2013-07-04

**Authors:** Irina Lonskaya, Michaeline L Hebron, Nicole M Desforges, Alexander Franjie, Charbel E-H Moussa

**Affiliations:** Department of Neuroscience, Laboratory for Dementia and Parkinsonism, Georgetown University Medical CenterWashington DC, USA

**Keywords:** β-amyloid, autophagy, parkin, Tau phosphorylation, tyrosine kinase

## Abstract

Tyrosine kinase inhibitors (TKIs) are effective therapies for leukaemia. Alzheimer is a neurodegenerative disease characterized by accumulation of β-amyloid (plaques) and hyper-phosphorylated Tau (tangles). Here we show that AD animals have high levels of insoluble parkin and decreased parkin-Beclin-1 interaction, while peripheral administration of TKIs, including Nilotinib and Bosutinib, increases soluble parkin leading to amyloid clearance and cognitive improvement. Blocking Beclin-1 expression with shRNA or parkin deletion prevents tyrosine kinase (TK) inhibition-induced amyloid clearance, suggesting that functional parkin-Beclin-1 interaction mediates amyloid degradation. Isolation of autophagic vacuoles (AVs) in AD mouse brain shows accumulation of parkin and amyloid, consistent with previous results in AD brains, while Bosutinib and Nilotinib increase parkin-Beclin-1 interaction and result in protein deposition in the lysosome. These data suggest that decreased parkin solubility impedes parkin-Beclin-1 interaction and amyloid clearance. We identified two FDA-approved anti-cancer drugs as potential treatment for AD.

Two FDA-approved tyrosine kinase inhibitor drugs, Bosutinib and Nilotinib, are shown to ameliorate Alzheimer's disease pathology in mouse models by increasing soluble parkin and leading to amyloid clearance and cognitive improvement.

## INTRODUCTION

Available tyrosine kinase inhibitors (TKIs), such as Imatinib, are effective in many patients with Philadelphia chromosome-positive chronic myelogenous leukaemia (CML) in chronic phase (de Lavallade et al, 2008; Kantarjian et al, 2007). Nilotinib (AMN107) is a second generation selective Bcr-Abl (Abelson) inhibitor, which is effective following Imatinib resistance and intolerance (Kantarjian et al, 2007). Nilotinib was approved by the US Food and Drug Administration (FDA) in 2007 for CML treatment (300–400 mg orally twice daily) (Deremer et al, 2008; Mahon et al, 2008; Skorski, 2011). Because Src and Abl are structurally homologous, Src inhibitors can also inhibit Abl (Musumeci et al, 2012). The dual Src/Abl TKI Bosutinib (SKI-606) was also approved by the FDA in 2012 (500 mg/kg orally once daily) (Cortes et al, 2011; Keller & Brummendorf, 2012; Puttini et al, 2006). Whereas Nilotinib is derived from Imatinib and binds to the same inactive conformation of Bcr-Abl (Weisberg et al, 2005), Bosutinib is active in CML after Imatinib or Nilotinib therapy failure (Khoury et al, 2012). Abl activation inhibits the E3 ubiquitin ligase activity of parkin, and Abl inhibition induces parkin protective function in Parkinson's disease (PD) models (Imam et al, 2011). Parkin is inactivated in the nigrostriatum of post-mortem sporadic PD patients (Ko et al, 2010; Lonskaya et al, 2012a), and decreased parkin solubility is associated with defects in autophagic clearance of β-amyloid and p-Tau in post-mortem Alzheimer's disease (AD) brains (Lonskaya et al, 2012c). Parkin mediates autophagic degradation of defective mitochondria (mitophagy) (Geisler et al, 2010; Narendra et al, 2008; Park et al, 2009; Vives-Bauza et al, 2010), and clears autophagic vacuoles (AVs) in AD and PD models (Khandelwal et al, 2011; Lonskaya et al, 2012a; Lonskaya et al, 2012c), while parkin deletion exacerbates amyloid pathology in AD models (Perucho et al, 2010; Rodriguez-Navarro et al, 2008).

AD is an aging disorder characterized by deposition of extracellular β-amyloid (Aβ) plaques and intraneuronal tangles containing hyper-phosphorylated Tau (p-Tau) (Hardy & Selkoe, 2002). Amyloid precursor protein (APP) is sequentially cleaved to yield C-terminal APP fragments (CTFs), and amyloid peptides Aβ_1–40_ and Aβ_1–42_ (Cook et al, 1997; Greenfield et al, 1999; Skovronsky et al, 1998; Xu et al, 1997) that give rise to extracellular plaques (D'Andrea et al, 2001; Gouras et al, 2000; Li et al, 2007; Oddo et al, 2003). Abl is associated with neuritic plaques and neurofibrillary tangles (NFTs) in AD (Derkinderen et al, 2005; Jing et al, 2009; Schlatterer et al, 2011; Tremblay et al, 2010). Abl phosphorylation at tyrosine 412 (T412) is elevated in the hippocampus and entorhinal cortex in AD post-mortem brains (Schlatterer et al, 2011; Tremblay et al, 2010). Src tyrosine kinase (TK) is also recognized in AD pathology via interaction with Tau (Ittner et al, 2010; Lee, 2005; Reynolds et al, 2008). Abl inhibition prevents Aβ_1–42_ fibrils and hydrogen peroxide (H_2_O_2_)-induced cell death (Alvarez et al, 2004), and hippocampal injection of Aβ fibrils leads to an increase of Abl levels (Cancino et al, 2008). These data led to the hypothesis that TKIs will activate parkin and facilitate autophagic amyloid clearance, thus limiting cell death and preventing cognitive decline in AD models. We used several TKIs, including Bosutinib and Nilotinib, which penetrate the brain and facilitate autophagy in AD models. The current studies evaluated the effects of TKIs Nilotinib and Bosutinib on parkin-mediated autophagic amyloid clearance in AD models.

## RESULTS

### Tyrosine kinase inhibition restores parkin-Beclin-1 interaction

**Figure 1:**
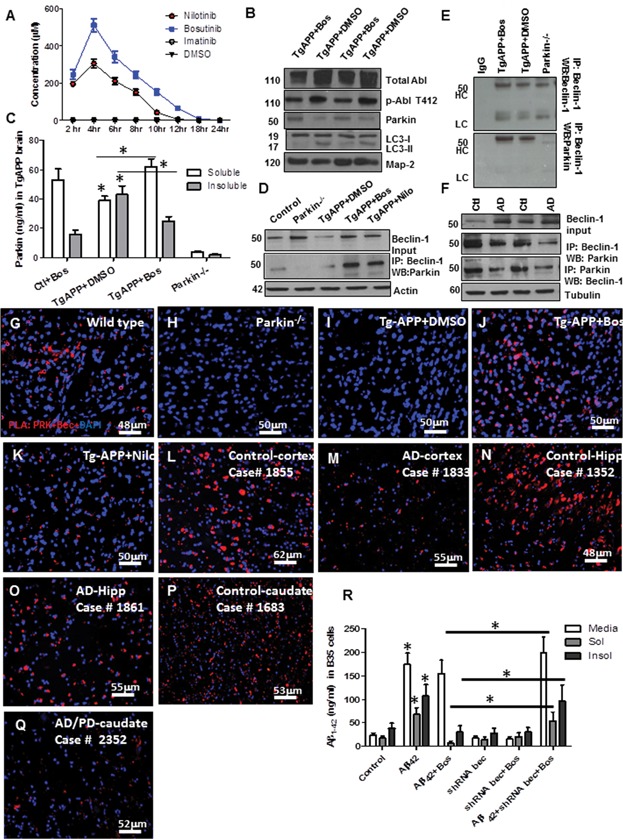
**Tyrosine Kinase inhibition restores parkin-Beclin-1 interaction**Graph represents brain levels of TKIs over a 24 hr period after IP injection with Imatinib, Nilotinib, Bosutinib and DMSO (*N* = 7). [Correction added after publication on 4 July 2013: The y axis of the graph shown in Fig. 1A has been corrected from “concentration (mM)” to “concentration (nM)”]WB in Tg-APP total brain lysates on 4–12% NuPAGE SDS gel (Invitrogen) show total Abl (top blot) T412 Abl (2^nd^ blot), soluble parkin level (3^rd^ blot), and LC3 (4^th^ blot) relative to MAP-2 (*N* = 9).Quantitative ELISA showing soluble (STEN extract) and insoluble (4 M urea extract) mouse parkin in Tg-APP (*N* = 9). Parkin^−/−^ brains were used as a specificity control.(D) Blots represent immunoprecipitated Beclin-1 in mice probed with parkin antibody, and (E) control IgG in parallel with immunoprecipitates, (F) immunoprecipitated Beclin-1 probed with parkin and the reverse experiment in post-mortem human AD cortex analyzed on 4–12% SDS-NuPAGE gel.*In situ* proximity ligation assay (PLA) shows endogenous parkin-Beclin-1 complexes in (G) WT C57BL/6 mice (*N* = 5) and (H) parkin^−/−^ as control.PLA in Tg-APP mice IP injected once daily for 3 weeks with (I) DMSO (J) 5 mg/kg Bosutinib and (K) 10 mg/kg Nilotinib (*N* = 5).PLA in human post-mortem brains in the (L) cortex of a normal subject and (M) cortex of an AD patient; the hippocampus of (N) a normal subject and (O) an AD patient; the caudate of (P) a normal subject and (Q) an AD patient.Graph represents human Aβ_1–42_ ELISA in rat B35 neuroblastoma cells transfected with human cDNA Aβ_1–42_ (or LacZ) or Beclin-1 shRNA for 24 hr, and then treated with 1 μM Bosutinib for an additional 24 hr (*N* = 12). *Significantly different to control or as indicated, Mean ± SEM, ANOVA with NeumannKeuls multiple comparison.

**Figure 2:**
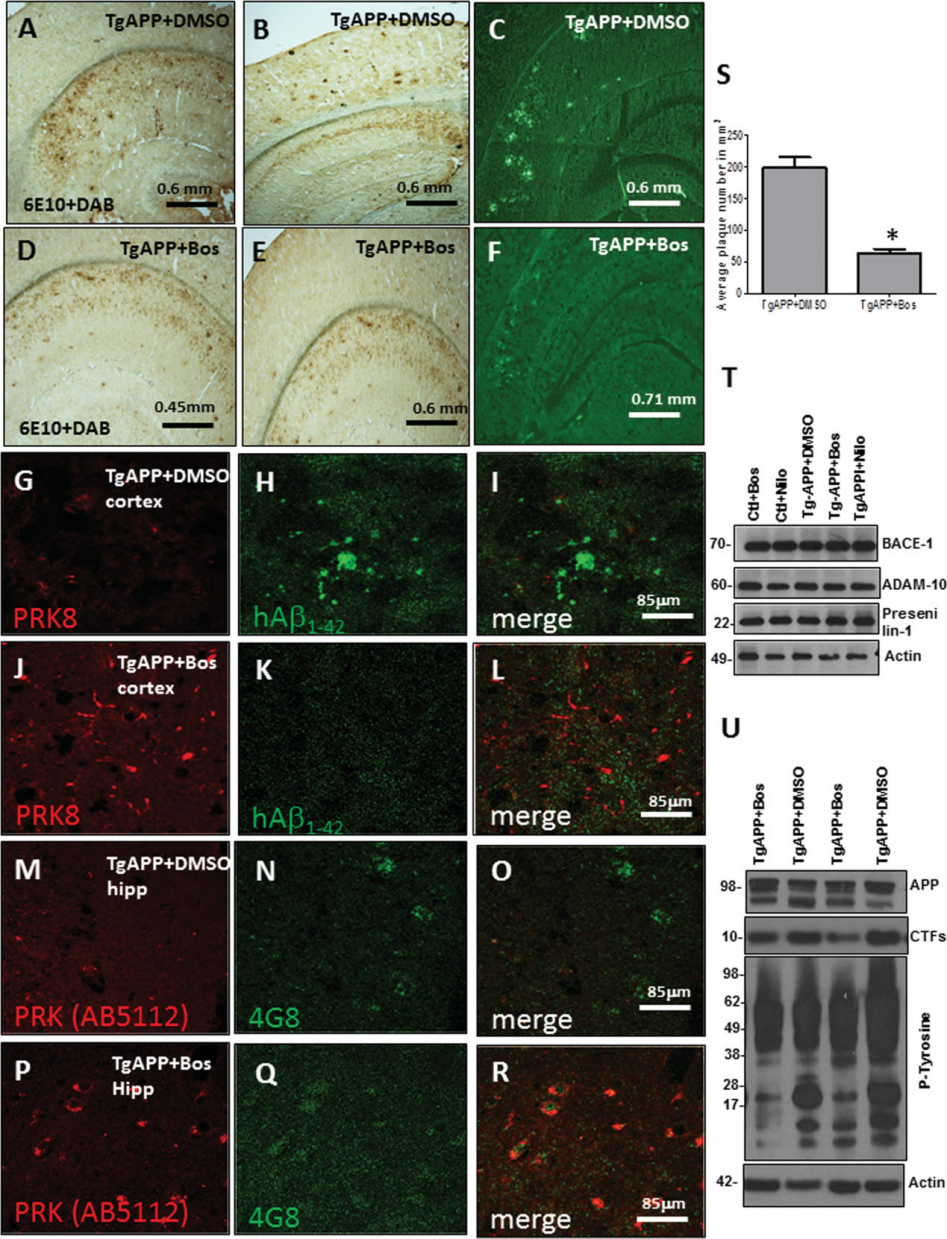
**Bosutinib decreases Aβ levels and eliminates plaques in AD models**Staining of 20 μm brain sections shows plaque formation with 6E10 antibody and DAB in the brain in different (A,B) Tg-APP + DMSO and (C) thioflavin-S staining in Tg-APP treated with DMSO (*N* = 7). (D,E) Tg-APP treated with 5 mg/kg Bosutinib for 3-weeks and (F) thioflavin-s staining.Staining of 20 μm thick brain sections shows (G) parkin, (H) Aβ_1–42_ and (I) merged figure in cortex of Tg-APP mice after 3 weeks of DMSO treatment, and (J) parkin, (K) Aβ_1–42_ and (L) merged figure in cortex of Tg-APP mice after 3 weeks of 5 mg/kg Bosutinib treatment. (M) Parkin, (N) Aβ_1–42_ and (O) merged figure in hippocampus of Tg-APP mice after 3 weeks of DMSO treatment, and (P) parkin, (Q) Aβ_1–42_ and (R) merged figure in cortex of Tg-APP mice after 3 weeks of Bosutinib treatment (*N* = 7).(S) Graphs represent quantification of amyloid plaques in Tg-APP with and without Bosutinib. WB in Tg-APP total brain lysates on 4–12% NuPAGE SDS gel (Invitrogen) showing (T) levels of BACE1 (1^st^ blot), ADAM-10 (2^nd^ blot) and presinilin-1 (3^rd^ blot) relative to actin and (U) total APP (top blot), CTFs (2^nd^ blot) and phospho-tyrosine (3^rd^ blot) relative to actin (*N* = 9).

To determine whether TKIs enter the brain, we intraperitoneally (IP) injected 2 months old C57BL6 mice with 10 mg/kg Imatinib, 10 mg/kg Nilotinib and 5 mg/kg Bosutinib in 30 μL DMSO and sacrificed the animals 2–24 h post-injection. No Imatinib (Gleevec) was detected by mass spectrometry in the brain compared to DMSO (Fig 1A), but Nilotinib reached a peak at 305 nM at 4 h and completely disappeared 12 h post-injection. Bosutinib, however, reached a peak at 510 nM 4 h post-injection and was still detectable 12 h (64 nM) post-injection (Fig 1A). [Correction added after publication on 4 July 2013: In the previous sentence, the concentrations have been corrected from μM to nM] No drugs were detected in the brain 18–24 h post-injection. Therefore, we chose Bosutinib as the main TKI in these studies. To determine the effects of Bosutinib on Abl inhibition and parkin levels *in vivo*, we treated (5 mg/kg IP injection) for 3 weeks 8–12 months old AD transgenic mice, which express neuronally derived human *APP* gene, 770 isoform, containing the Swedish K670N/M671L, Dutch E693Q and Iowa D694N mutations (Tg-APP) under the control of the mouse thymus cell antigen 1, theta, *Thy1*, promoter (Davis et al, 2004). Western blot (WB) showed 30% decrease in total Abl (Fig 1B, 71 ± 13, mean ± sd, *p* = 0.04, *n* = 9) and 43% (57 ± 13, mean ± sd) T412 Abl relative to MAP-2 control (100 ± 11, mean ± sd, *p* = 0.04), suggesting that Bosutinib inhibits Abl phosphorylation (activity). Bosutinib decreased the level of T412 Abl (125 ± 29, mean ± sd, *p* = 0.04) compared to total Abl. Soluble parkin (STEN extract, supernatant) was significantly increased (128 ± 32, mean ± sd, *p* = 0.04) relative to MAP-2 (Fig 2B). Because TKIs are reported to alter autophagy (Salomoni & Calabretta, 2009), the level of light chain protein (LC)-3 was examined. No changes were detected in LC3-I levels compared to MAP-2 in Tg-APP mice (Fig 1B), but LC3-II, which indicates the amount of autophagosomes, was detected and Bosutinib significantly decreased LC3-II relative to both LC3-I (16 ± 2.4, mean ± sd, *p* = 0.0001) and MAP-2 (29 ± 8, mean ± sd, *p* = 0.0001) levels (Fig 1B, *N* = 9), suggesting that Abl inhibition increases parkin and facilitates autophagic clearance. Quantitative ELISA, using parkin^−/−^ brains as specificity controls (Fig 1C) showed a significant decrease in soluble (STEN extract) parkin in Tg-APP (39 ng/mL) compared to age-matched (53 ng/mL) control (Fig 1C, *p* = 0.04, *N* = 9) and Bosutinib reversed parkin level above control (62 ng/mL), consistent with the WB results. However, insoluble (4 M urea extract from pellet) parkin in Tg-APP was increased (43 ng/mL) compared to age-matched (16 ng/mL) control (Fig 1C, *p* = 0.04, *N* = 9) and Bosutinib reversed insoluble parkin slightly above control (25 ng/mL).

Beclin-1 levels were increased (144 ± 37, mean ± sd, *p* < 0.02, *N* = 7) relative to actin in parkin^−/−^ mice compared to control (Fig 1D, first blot) and in Tg-APP mice treated with Bosutinib (138 ± 42, mean ± sd, *p* = 0.02) and Nilotinib (144 ± 43, mean ± sd, *p* = 0.02) relative to actin (*N* = 7). There was no difference in Beclin-1 between DMSO treated control and Tg-APP mice relative to actin. Beclin-1 was immunoprecipitated from wild type, parkin^−/−^ and Tg-APP mice (Fig 1D, first blot is input) and probed with parkin. As expected no Beclin-1-parkin interaction was detected in parkin^−/−^ compared to wild type mice (Fig 1D, second blot, *N* = 5), and this interaction was decreased in Tg-APP mice treated with DMSO, but stronger parkin bands were detected with Bosutinib and Nilotinib, suggesting increased interaction between parkin and Beclin-1. Control experiments were conducted using Beclin-1 immunoprecipitation from brain lysates and analysed by WBs with anti-Beclin-1 (Fig 1E, top blot) and anti-parkin (bottom blot) antibodies along with IgG control and parkin^−/−^. HRP-secondary antibodies showed no bands in IgG lanes and no parkin in Parkin^−/−^ mice, indicating specificity of Beclin-1 and parkin bands. Beclin-1 was also increased (Fig 1F, 161 ± 48, mean ± sd, *N* = 5, *p* = 0.01) in post-mortem AD cortex relative to tubulin compared to control subjects. Significantly decreased levels were detected when immunoprecipitated Beclin-1 was probed with parkin (Fig 1F, 28 ± 6.5, mean ± sd, *p* < 0.03, *N* = 5) and inversely when immunoprecipitated parkin was probed with Beclin-1 (46 ± 18, mean ± sd, *N* = 5), suggesting decreased parkin-Beclin-1 interaction in AD.

We previously reported decreased parkin solubility and autophagic defects in post-mortem AD (Lonskaya et al, 2013) and PD (Lonskaya et al, 2012a) brains, and autophagic alterations were reversed with lentiviral parkin, which increased Beclin-1 levels. We confirmed the interaction between parkin and Beclin-1 using *in situ* proximity ligation assay (PLA), which allows direct observation of individual endogenous protein complexes (Soderberg et al, 2006). Parkin-Beclin-1 interaction was observed in C57BL/6 mice (Fig 1G) compared to parkin^−/−^ (Fig 1H). Interestingly, no parkin-Beclin-1 interaction was detected in Tg-APP mice (Fig 1I, *N* = 5), but 5 mg/kg Bosutinib (Fig 1J) or 10 mg/kg Nilotinib (Fig 1K) led to parkin-Beclin-1 interaction (*N* = 5). These results were confirmed in human post-mortem brains (described in (Lonskaya et al, 2012a; Lonskaya et al, 2013)), which showed noticeable interaction in the cortex (Fig 1L), hippocampus (Fig 1N) and caudate (Fig 1P), but this was decreased (*N* = 7 control and 12 AD) in AD cortex (Fig 1M) and Hippocampus (Fig 1O), and caudate (Fig 1Q), reflecting alteration of parkin solubility in these brains. To ascertain that Beclin-1 mediates Bosutinib effects on amyloid clearance, rat B35 neuroblastoma cells were transfected with 3 μg human cDNA Aβ_1–42_ (or LacZ) or Beclin-1 shRNA for 24 h, and then treated with 1 μM Bosutinib for an additional 24 h. Aβ_1–42_ levels were unaffected in the media with Bosutinib compared to Aβ_1–42_ expressing cells (Fig 1R, *N* = 12). Bosutinib decreased soluble (STEN extract supernatant) and insoluble (30% formic acid from pellet) Aβ_1–42_ (81 and 67%, *p* = 0.012, respectively) compared to Aβ_1–42_ transfected cells. Blocking Beclin-1 expression prevented efficient clearance of Aβ_1–42_, which accumulated in the media and slightly decreased in cellular fractions compared to Aβ_1–42_ alone, indicating that Beclin-1 is required for complete Aβ_1–42_ clearance. Soluble and insoluble Aβ_1–42_ were significantly higher when Beclin-1 was blocked compared to Bosutinib in Aβ_1–42_-expressing cells (Fig 1R).

### Bosutinib decreases Aβ levels and reduces plaques in AD models

Staining of 20 μm brain sections shows plaque formation in Tg-APP mice treated with DMSO (Fig 2A, B representing different animals, *N* = 7), confirmed by thioflavin-S staining (Fig 2C), though plaque staining was reduced in the Bosutinib group after 3-week treatment (Fig 2D–F). Higher magnification shows endogenous parkin staining in Tg-APP (Fig 2G) and plaque deposition (Fig 2H and I) in the hippocampus. Bosutinib increased endogenous parkin (Fig 2J) and reduced plaque load (Fig 2K and L). Using different parkin antibodies to show parkin (Fig 2M) and plaques (Fig 2N and O), Bosutinib increased parkin levels (Fig 2P) and reduced plaques (Fig 2Q and R) in the cortex. Quantification of plaque load in Tg-APP mice was performed by a blind investigator using ImageJ by drawing a line around individual plaques within 1 mm^2^ radius of 6 randomly selected hippocampal and cortical regions in 6E10 stained slides (*N* = 9). The average number of plaques was significantly reduced in Bosutinib (Fig 2S, 64 ± 10, mean ± sd per mm^2^, *p* = 0.02, *N* = 9) compared to DMSO (Fig 2S, 198 ± 49, mean ± sd per mm^2^, *p* = 0.02, *N* = 9) treated mice. WB analysis showed no difference between control and Tg-APP mice with either Bosutinib or Nilotinib and DMSO (WT data not shown) in the level of APP cleavage enzymes (Fig 2T, *N* = 4), including β-secretase (BACE-1, first blot), α-secretase (ADAM-10, second blot) and γ-secretase (Presenilin-1, third blot), suggesting that TKI-induced decrease in Aβ levels is unlikely to be mediated by changes of expression of APP-cleaving secretases. No change in total APP was detected but CTFs were decreased (Fig 2U, 74 ± 21, mean ± sd, *p* = 0.04, *N* = 9) in Tg-APP + Bosutinib compared to DMSO (100 ± 36, mean ± sd). We further examined whether the reduction of Aβ level in Tg-APP mice was due to clearance mechanisms via lentiviral injection of Aβ_1–42_ that is not derived from APP cleavage (Fig 4). Bosutinib also decreased phospho-tyrosine proteins level (Fig 2U), indicating that it is not a specific Src-Abl inhibitor.

### Chronic treatment with TKI alters brain amyloid level

**Figure 3:**
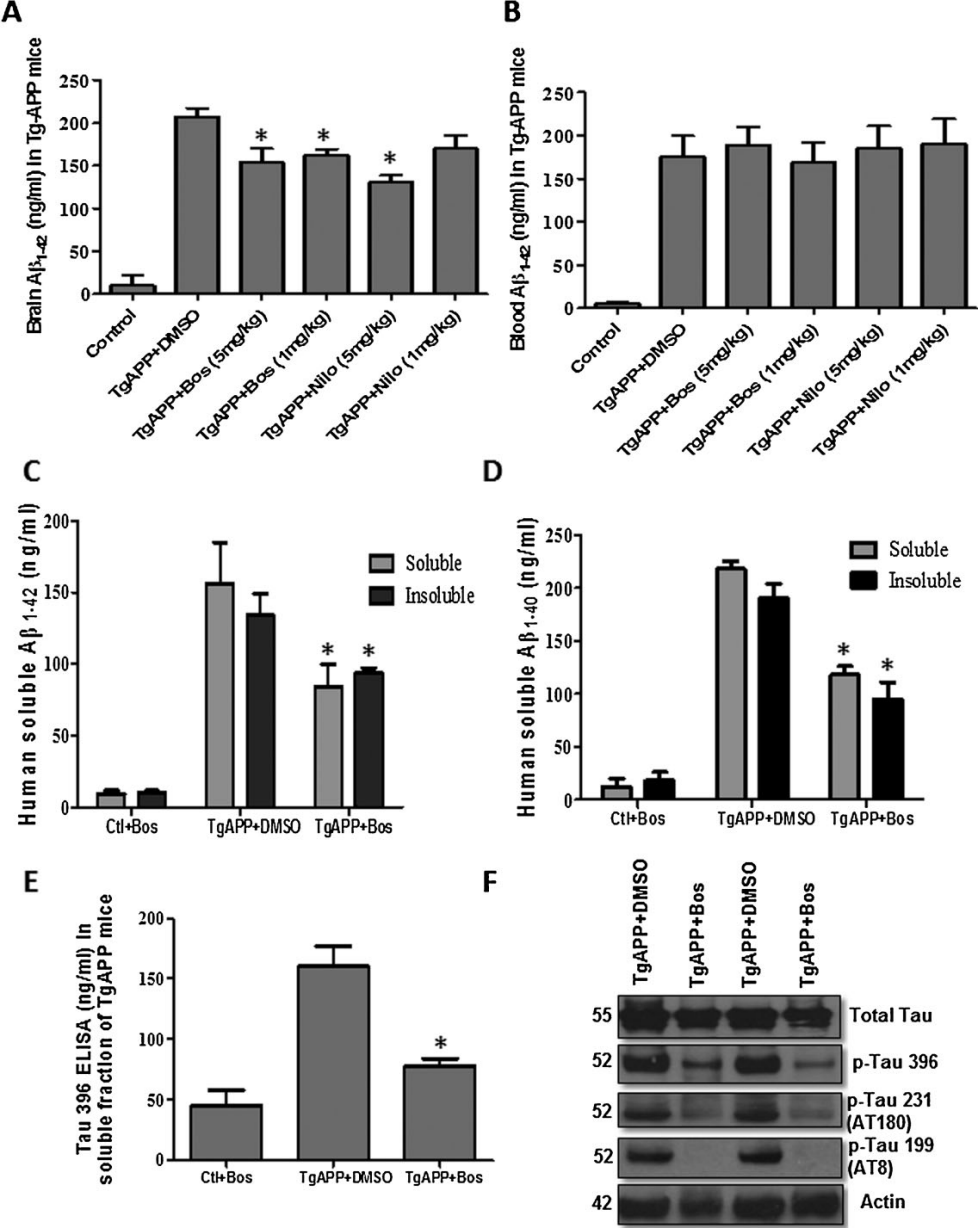
Chronic treatment with TKI alters brain amyloid level*Significantly different to control, Mean ± SEM, ANOVA with Neumann Keuls multiple comparison, *p* < 0.05.Graphs represent ELISA of human Aβ_1–42_ in (A) brain levels and (B) blood Aβ_1–42_ levels in 8-month old Tg-APP mice injected I.P. every other day for 6 weeks with Bosutinib or Nilotinib (*N* = 10).ELISA of human soluble and insoluble brain (C) Aβ_1–42_ and (D) Aβ_1-40_ levels as well as (E) mouse p-Tau levels in 8-month old Tg-APP mice injected IP daily for 3 weeks with 5 mg/kg Bosutinib (*N* = 9).WB in Tg-APP total brain lysates on 4–12% NuPAGE SDS gel (Invitrogen) show total Tau (1^st^ blot), serine 396 p-Tau (2^nd^ blot), threonine 231 (AT180, 3^rd^ blot) and serine 199 p-Tau (AT8, 4^th^ blot) relative to actin (*N* = 7).

TKIs are pleiotropic drugs that affect a wide range of phospho-tyrosine proteins; therefore, a lower dose may prevent some of the off-site effects of the drugs. IP injection every second day for 6 weeks resulted in significant reductions in brain Aβ_1–42_ levels in 8-month old Tg-APP mice with 5 mg/kg (154 ng/mL) or 1 mg/kg (162 ng/mL) Bosutinib compared to DMSO (207 ng/mL) treated mice (Fig 3A, *p* < 0.03, *N* = 10). Nilotinib (5 mg/kg) also reduced Aβ_1–42_ (131 ng/mL) in the brain (Fig 3A, *p* = 0.03, *N* = 10) but lower dose (1 mg/kg) did not have any effects. High levels of Aβ_1–42_ (175 ng/mL) was detected in the blood (Fig 3B, *p* = 0.04, *N* = 10) in Tg-APP mice compared to control, but drug treatment did not change this level. However, daily 5 mg/kg Bosutinib injection for 3 weeks (Fig 3C) resulted in a significant decrease of soluble (STEN extract) Aβ_1–42_ (84 ng/mL, *p* = 0.015, *N* = 9) compared to DMSO (150 ng/mL) and insoluble (30% formic acid) Aβ_1–42_ (94 ng/mL) compared to DMSO (134 ng/mL). Bosutinib (5 mg/kg daily for 3 weeks) also reduced soluble (190 ng/mL) and insoluble (94 ng/mL) Aβ_1–40_ (Fig 3D, *p* = 0.03, *N* = 9) compared to DMSO (134 and 134 ng/mL, respectively) as well as serine 396 p-Tau (Fig 3E, 78 ng/mL, *p* = 0.03, *N* = 9) compared to DMSO (160 ng/mL). WB showed a significant decrease (80 ± 24, mean ± sd) in total Tau (Fig 3F, *p* = 0.03, *N* = 7) relative to actin, and significant decreases of serine-396 (36 ± 9, mean ± sd, *p* = 0.01), threonine-231 (30 ± 8.4, mean ± sd, *p* = 0.01, AT180) and elimination of serine-199 (AT8) p-Tau relative to total Tau compared to DMSO (100 ± 31, mean ± sd, *p* = 0.0001). No phospho-tyrosine Tau was detected with the commercially available antibody (4G10, Millipore) and immunoprecipitation of total Tau (Tau-5 antibody) and probing with total phospho-tyrosine did not show any difference between control and Tg-APP mice (data not shown), suggesting that Tau phosphorylation at Ser and Thr may affect phosphorylation at tyrosine residues at later stages of Tau pathology.

### Bosutinib degrades lentiviral Aβ_1–42_ in a parkin-dependent manner

**Figure 4:**
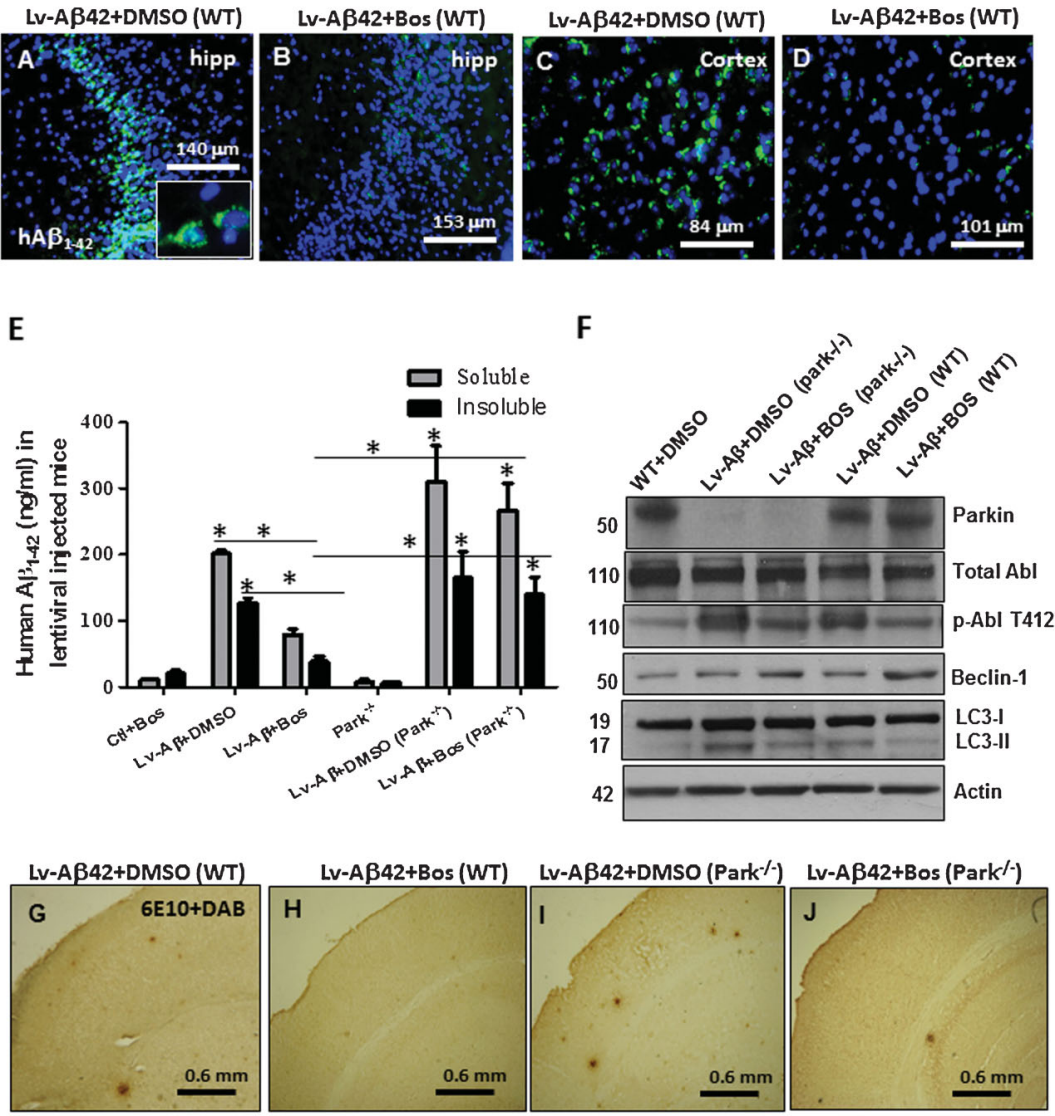
**Bosutinib degrades lentiviral Aβ_1–42_ in a parkin-dependent manner**Staining of 20μm brain sections shows intracellular Aβ_1–42_ within the (A) hippocampus of lentiviral Aβ_1–42_ injected WT mice, and (B) Bosutinib clearance of intracellular Aβ_1–42_. Staining of 20μm brain sections shows intracellular Aβ_1–42_ within the (C) cortex of WT mice with the lentiviral Aβ_1–42_, and (D) Bosutinib clearance of intracellular Aβ_1–42_ (*N* = 7).ELISA of human soluble and insoluble Aβ_1–42_ at 6 weeks post-injection of lentiviral Aβ_1–42_ and daily treatment with 5 mg/kg Bosutinib (*N* = 9) for 3 weeks. * Significantly different to control or as indicated, Mean ± SEM, ANOVA with Neumann Keuls multiple comparison.WB on 4–12% NuPAGE SDS gel of total brain lysates in 1 year old wild type and parkin^−/−^ mice treated with 5 mg/kg Bosutinib for 3 weeks on 4–12% NuPAGE SDS gel (Invitrogen) showing parkin (1^st^ blot)total Abl (2^nd^ blot), T412 Abl (3^rd^ blot), Beclin-1 (4th blot) and LC3 (5^th^ blot) relative to actin (*N* = 7).Staining of 20μm brain sections shows plaque formation with 6E10 antibody and DAB 6 weeks post-injection with (G) lentiviral Aβ_1–42_ + 3 weeks DMSO treatment and (H) IP injection with 5 mg/kg Bosutinib 3 weeks post-lentiviral expression (3 weeks treatment) clears plaques in WT mice (*N* = 7).(I) Lentiviral Aβ_1–42_ + DMSO and (J) IP injection with 5 mg/kg Bosutinib 3 weeks post-lentiviral expression (3 weeks treatment) in parkin^−/−^ mice (*N* = 7).

We stereotaxically injected 1 × 10^6^ multiplicity of infection (m.o.i) lentiviral Aβ_1–42_ bilaterally into the hippocampus of 1 year old C57BL/6 and parkin^−/−^ mice and 3 weeks later we injected (IP) 5 mg/kg Bosutinib once a day for 3 additional weeks. Lentiviral injection showed intracellular Aβ_1–42_ within the hippocampus (Fig 4A, *N* = 7) and Bosutinib clearance of Aβ_1–42_ (Fig. 4B). Lentiviral injection into the hippocampus led to Aβ_1–42_ expression throughout the cortex (Fig 4C) and Bosutinib eliminated Aβ_1–42_ accumulation (Fig 4D). Human Aβ_1–42_ ELISA showed high levels of soluble (201 ng/mL) and insoluble (126 ng/mL) Aβ_1–42_ compared in WT lentiviral Aβ_1–42_ expressing mice compared to control (Fig 4E, *p* = 0.0001, *N* = 9), while Bosutinib reduced soluble (126 ng/mL) and insoluble (38 ng/mL) Aβ_1–42_. Soluble (310 ng/mL) and insoluble (165 ng/mL) Aβ_1–42_ were also increased in lentiviral Aβ_1–42_ expressing parkin^−/−^ mice compared to control and WT Aβ_1–42_ expressing mice (Fig 4E, *p* = 0.001, *N* = 9). Bosutinib did neither reduce soluble (165 ng/mL) nor insoluble (140 ng/mL) Aβ_1–42_ in parkin^−/−^ compared to WT, further suggesting a role for endogenous parkin in Aβ_1–42_ clearance. WB analysis of total brain lysates (Fig 4F) showed significantly increased parkin (142 ± 41, mean ± sd, *p* = 0.04, *N* = 7) levels relative to actin in Bosutinib treated mice compared to DMSO (100 ± 31, mean ± sd) with and without Aβ_1–42_. A non-significant difference (16%) in total Abl levels were detected but T412 Abl was significantly decreased (Fig 4F, *p* = 0.02, *N* = 7) relative to total Abl (52 ± 13, mean ± sd) or actin (48 ± 12, mean ± sd) with Bosutinib treatment compared to DMSO (100 ± 24, mean ± sd) in WT and parkin^−/−^ mice. An increase in Beclin-1 (32%) relative to actin (Fig 4F, *p* = 0.04, *N* = 7) was detected in parkin^−/−^ mice and Beclin-1 increased (144 ± 51, mean ± sd) with Bosutinib relative to actin compared to WT + DMSO mice (100 ± 29, mean ± sd). No significant differences in LC3-I levels were observed relative to actin (Fig 4F), but LC3-II appeared in Aβ_1–42_ expressing WT and parkin^−/−^ mice compared to control. However, Bosutinib did not affect the level of LC3-II relative to LC3-I and actin in parkin^−/−^ mice, but reduced LC3-II level relative to actin (59 ± 18, mean ± sd) and LC3-I (69 ± 22, mean ± sd) in WT mice (Fig 4F, 100 ± 34, mean ± sd, *p* = 0.039, *N* = 7). Staining with 6E10 antibody 6 weeks post-injection with lentiviral Aβ_1–42_ (+3 weeks DMSO) showed plaque formation in Aβ_1–42_ expressing mice (Fig 4G), and daily 5 mg/kg Bosutinib for 3 weeks reduces plaques (Fig 4H, *N* = 7). Aβ_1–42_ expression in parkin^−/−^ mice showed more plaques (Fig 4I) and Bosutinib did not reduce plaques in these mice (Fig 4J), suggesting that Aβ_1–42_ clearance depends on parkin expression.

### Autophagic amyloid clearance is impaired in the absence of parkin

**Figure 5:**
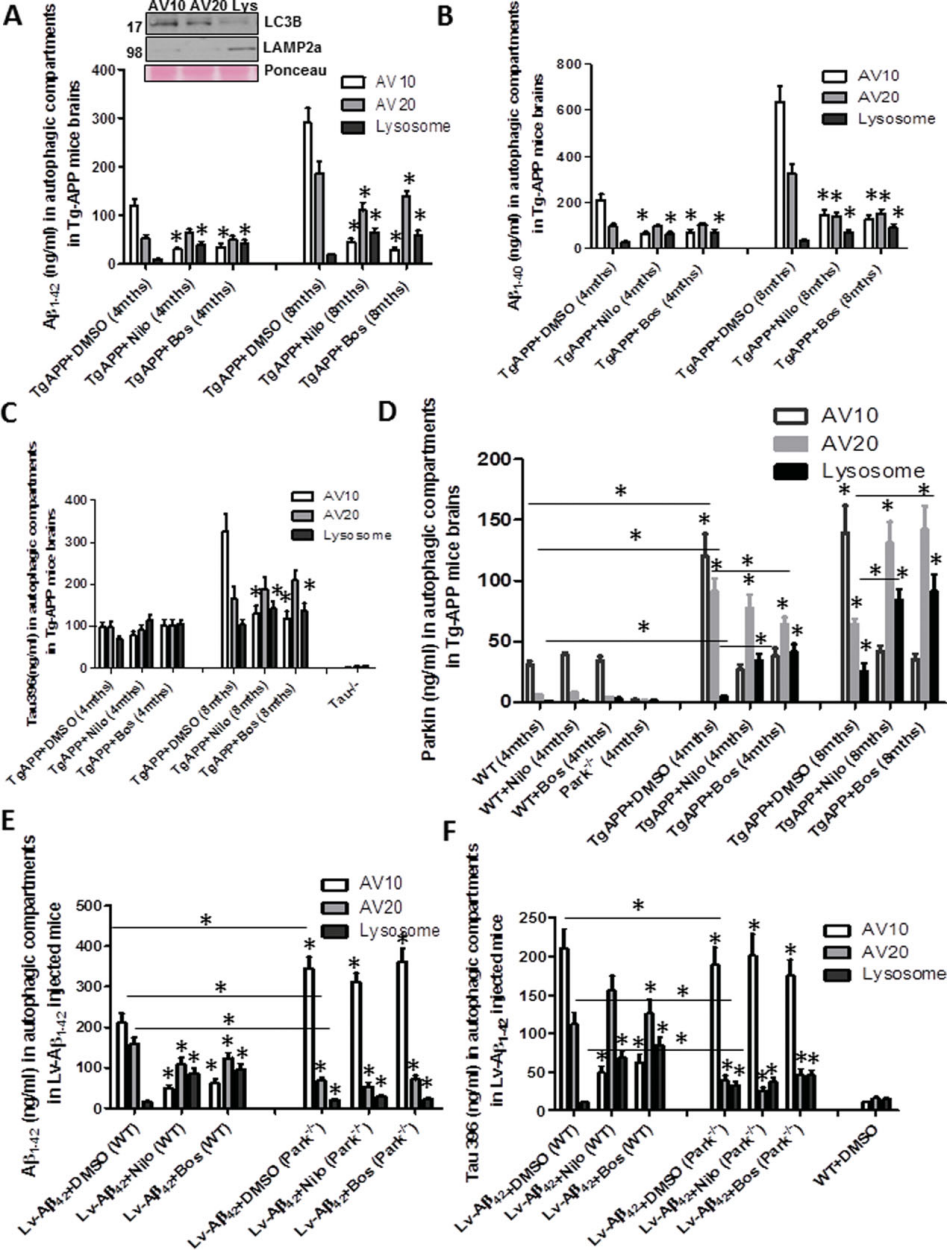
Subcellular fractionation suggests impaired autophagy in the absence of parkin*Significantly different to control or as indicated, Mean ± SEM, ANOVA with Neumann Keuls multiple comparison.Graphs represent ELISA (*N* = 5) inautophagic vacuoles (AVs) in the brain of 4 and 8 months old Tg-APP mice treated with 10 mg/kg Nilotinib or 5 mg/kg Bosutinib (*N* = 5) for 3 weeks showing (A)human Aβ_1–42_ (Insert is WB analysis of AVs showing LC3-B in AV10 and AV20 (1^st^ blot) and LAMP2a in Lys (2^nd^ blot) fraction) and (B)Aβ_1-40_,(C) p-Tau and (D) parkin.ELISA in autophagic vacuoles of 1 year old WT and parkin^−/−^ mice (*N* = 5) injected with lentiviralAβ_1–42_ for 3 weeks and treated with 10 mg/kg Nilotinib and 5 mg/kg Bosutinib for 3 additional weeks showing (E) human Aβ_1–42_ and (F) p-Tau levels.

To ascertain that autophagy is involved in TKI-mediated amyloid clearance *in vivo*, AVs were isolated via subcelullar fractionation using a Metrizamide gradient, and autophagosomes were identified with LC3-B antibodies (Fig 5A, insert) in AV10 and AV20 and the lysosomal fraction was identified with lysosomal associated membrane protein (LAMP2a) as we previously described (Lonskaya et al, 2012c). Aβ_1–42_ was detected by ELISA in AV10 (120 ng/mL) and AV20 (52 ng/mL) in 4 months old Tg-APP (Fig 5A, *N* = 5) but Aβ_1–42_ was decreased by 10 mg/kg Nilotinib (30 ng/mL, *p* = 0.001) and 5 mg/kg Bosutinib (35 ng/mL, *p* = 0.03) in AV10. Nilotinib and Bosutinib increased Aβ_1–42_ levels in the lysosomes (40 ng/mL and 43 ng/mL, respectively). Aβ_1–42_ was even higher in AV10 (290 ng/mL) and AV20 (186 ng/mL) in 8 months old Tg-APP brain (Fig 5A, *p* = 0.0001, *N* = 5) but Aβ_1–42_ was decreased by Nilotinib in AV10 (35 ng/mL) and AV20 (120 ng/mL) and Bosutinib in AV10 (29 ng/mL) and AV20 (39 ng/mL) compared to DMSO (*p* = 0.001). Nilotinib and Bosutinib increased Aβ_1–42_ levels in the lysosomes (65 and 59 ng/mL, respectively) compared to DMSO. Human Aβ_1–40_ was also detected in AV10 (211 ng/mL) and AV20 (95 ng/mL) in 4 months old Tg-APP brain (Fig 5B, *p* = 0.02, *N* = 5) and this level was decreased by Nilotinib (65 ng/mL) and Bosutinib (71 ng/mL) in AV10. Nilotinib and Bosutinib significantly increased Aβ_1–40_ levels (64 and 71 ng/mL, respectively) in the lysosomes. Aβ_1–40_ was also higher in AV10 (636 ng/mL) and AV20 (325 ng/mL) in 8 months old Tg-APP brain (Fig 5B, *p* = 0.00001, *N* = 5) and it was decreased by Nilotinib in AV10 (145 ng/mL) and AV20 (140 ng/mL) and Bosutinib in AV10 (126 ng/mL) and AV20 (130 ng/mL) compared to DMSO. Nilotinib and Bosutinib increased Aβ_1–40_ (71 and 89 ng/mL, respectively) in the lysosomes compared to DMSO (*p* = 0.001). p-Tau was detected in AV10 (325 ng/mL) and AV20 (165 ng/mL) in 8 months old Tg-APP brain (Fig 5C, *p* = 0.002, *N* = 5) and it was decreased in AV10 by Nilotinib (130 ng/mL) and Bosutinib (119 ng/mL) compared to DMSO. Nilotinib and Bosutinib increased p-Tau levels in the lysosomes (142 and 136 ng/mL, respectively) compared to DMSO (104 ng/mL).

No differences in parkin levels were detected in WT mice (Fig 5D, *N* = 5), using parkin^−/−^ as a negative control. Parkin was detected in AV10 (120 ng/mL) and AV20 (91 ng/mL) in 4 months old Tg-APP brain compared to WT (Fig 5D, *p* = 0.001, *N* = 5), but Nilotinib decreased parkin (27 ng/mL). Bosutinib decreased parkin (38 ng/mL) in AV10, and AV20 (77 and 64 ng/mL, respectively) compared to Tg-APP DMSO and increased it (38 and 42 ng/mL, respectively) in the lysosomes. Parkin was also increased in AV10 (139 ng/mL) and AV20 (64 ng/mL) in 8-months Tg-APP mice compared to WT (Fig 5D, *p* = 0.0001, *N* = 5). Nilotinib and Bosutinib decreased parkin in AV10 (42 and 35 ng/mL, respectively) and increased it in AV20 (131 and 142 ng/mL, respectively), and both increased parkin in the lysosomes (64 and 75 ng/mL, respectively) compared to DMSO.

To further determine the role of parkin in TKI-mediated autophagic clearance of amyloid, AVs were isolated from WT and parkin^−/−^ mice expressing lentiviral Aβ_1–42_ for 3 weeks and treated with 10 mg/kg Nilotinib or 5 mg/Kg Bosutinib for 3 additional weeks. Lentiviral injection led to Aβ_1–42_ accumulation in AV10 (211 ng/mL) and AV20 (159 ng/mL) in WT brain (Fig 5E, *N* = 5) and Nilotinib decreased Aβ_1–42_ in AV10 (49 ng/mL, *p* = 0.001, *N* = 5) and AV20 (109 ng/mL) and increased it (87 ng/mL) in lysosomes compared to DMSO. Bosutinib also decreased Aβ_1–42_ in AV10 (62 ng/mL, *p* = 0.0001, *N* = 5) and AV20 (124 ng/mL) and increased it (96 ng/mL) in lysosomes compared to DMSO. Aβ_1–42_ was significantly increased in AV10 (345 ng/mL) and reduced in AV20 (68 ng/mL) in lentiviral Aβ_1–42_ parkin^−/−^ mice compared to WT. However, Nilotinib and Bosutinib did not alter Aβ_1–42_ in AVs in lentiviral Aβ_1–42_ expressing parkin^−/−^, which remained higher than lentiviral Aβ_1–42_ WT mice (Fig 5E, *p* = 0.0001, *N* = 5). p-Tau was also detected in AV10 (211 ng/mL) and AV20 (112 ng/mL) in lentiviral Aβ_1–42_ expressing WT brain (Fig 5F) and Nilotinib decreased p-Tau in AV10 (49 ng/mL, *p* = 0.02, *N* = 5) and increased it in lysosomes (17 ng/mL) compared to DMSO. Similarly, Bosutinib decreased p-Tau in AV10 (62 ng/mL) and increased it in lysosomes (24 ng/mL) compared to DMSO. Higher p-Tau levels were detected in AV10 in parkin^−/−^ (189 ng/mL) compared to WT but p-Tau was decreased in AV20 (16 ng/mL) and increased in lysosomes (10 ng/mL) compared to WT (Fig 5F, *p* < 0.05, *N* = 5). TKI did not alter p-Tau levels in AVs compared to lentiviral Aβ_1–42_ expressing parkin^−/−^, and they all remained significantly higher than lentiviral Aβ_1–42_ WT mice (Fig 5E, *p* = 0.002, *N* = 5).

### Bosutinib improves cognitive performance in a parkin-dependent manner

**Figure 6:**
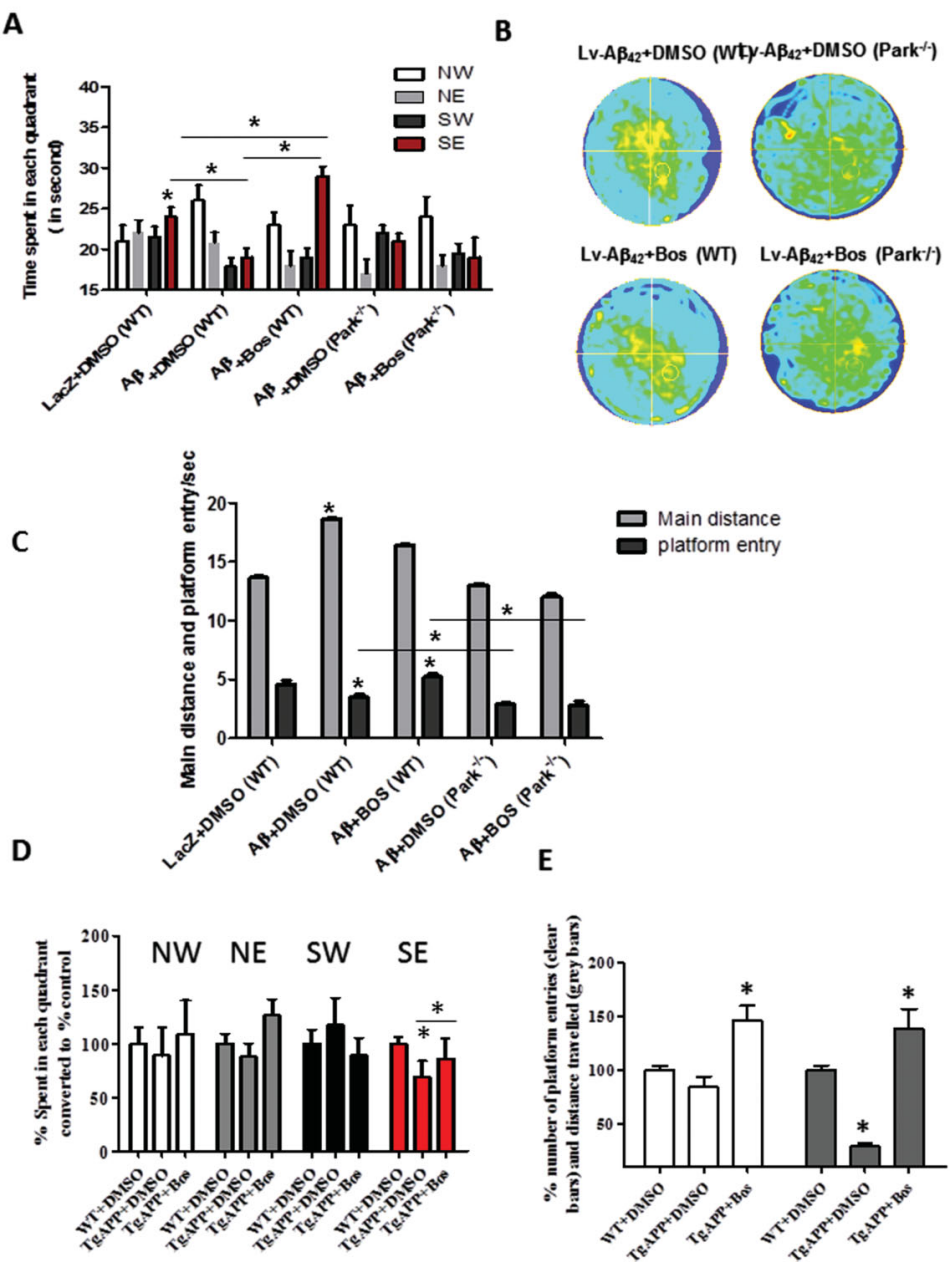
Bosutinib improves cognitive performance in a parkin-dependent manner*Significantly different to DMSO or as indicated, Mean ± SEM, ANOVA with Neumann Keuls multiple comparison.(A) Represents results of Morris water maze test in lentiviralAβ_1–42_-injected ± Bosutinib WT (*N* = 12) and parkin^−/−^ (*N* = 10) mice, and (B) heat maps for each group.Graphs represent total number of entry into platform area and distance travelled.Represents results of Morris water maze test in Tg-APP ± Bosutinib (*N* = 12) mice.Graphs represent total number of entry into platform area and distance travelled.

Cognition was assessed using Morris water maze. Aβ_1–42_-injected (+DMSO) mice spent less time (48% in seconds) in the SE quadrant (Fig 6A and B, *p* = 0.03, *N* = 12), where the training platform was originally placed, compared to WT (LacZ + DMSO), while Bosutinib increased time spent in SE area even more than WT (22%) and DMSO treated Aβ_1–42_ mice (2.1-fold). Aβ_1–42_ parkin^−/−^ mice (*N* = 10) did not show any significant changes compared to Aβ_1–42_-injected (+DMSO) or LacZ WT mice. A heat map for each group (Fig 6B) shows that Aβ_1–42_ (DMSO) mice spent more time roaming without reaching the SE area, while Bosutinib improved area search in WT mice (Fig 6B). In contrast, parkin^−/−^ ± Bosutinib did not reach the platform area (Fig 6B). WT Aβ_1–42_ mice entered the SE area less (Fig 6C, 23%) than WT, but Bosutinib increased the number of entries compared to Aβ_1–42_ mice with DMSO. Parkin^−/−^ entered less (39%, *p* = 0.04, *N* = 10). Aβ_1–42_ WT mice travelled (Fig 6C) significantly more distance than WT (21%). Main distance was not changed in parkin^−/−^ ± Bosutinib compared to control, suggesting that the changes in platform entry are not attributable to less movement in parkin^−/−^ mice. These experiments were repeated in 1 year old Tg-APP mice and age-matched control (C57BL/6). Tg-APP (+DMSO) mice reached the SE platform less than WT (Fig 6D, 54%, *p* = 0.04, *N* = 12) and Bosutinib significantly reversed time spent in SE (45%) compared to DMSO. The distance travelled (Fig 6E) was increased in Bosutinib (86%) compared to DMSO Tg-APP mice (clear bars), which had values 41% below control levels (Fig 6E, *p* = 0.02, *N* = 12). Platform entry was less in DMSO Tg-APP (76%) compared to WT (Fig 6E, full bars) and Bosutinib significantly increased platform entry (6.2-fold) compared to DMSO Tg-APP mice (Fig 6E, *p* = 0.03, *N* = 12).

## DISCUSSION

These studies evaluated the effects of TKI on parkin-Beclin-1 interaction and modulation of autophagic amyloid clearance in AD models. Here we show novel mechanisms of parkin-Beclin-1 interaction, which is dependent on parkin stability as increased levels of insoluble parkin in AD and Tg-APP mice lead to loss of parkin-Beclin-1 interaction, perhaps impairing autophagic amyloid clearance. These data, along with the identification of brain penetrant FDA-approved drugs, are novel mechanistic and translational findings. These results demonstrate the impact of decreased parkin solubility (Lonskaya et al, 2012b; Lonskaya et al, 2013), which co-localizes with intraneuronal Aβ_1–42_ in post-mortem AD brains (Lonskaya et al, 2012c), suggesting failure to facilitate amyloid clearance. We previously reported that exogenous parkin mediates autophagic clearance (Burns et al, 2009; Khandelwal et al, 2011; Lonskaya et al, 2012b; Lonskaya et al, 2013; Rebeck et al, 2010) and here we delineate the mechanisms related to parkin function via interaction with Beclin-1 to facilitate autophagosome maturation (Lonskaya et al, 2012b; Lonskaya et al, 2013), suggesting that parkin stability affects its protein clearance ability. Although the effects of osmotic pump delivery of TKIs on microgliosis (Dhawan & Combs, 2012), Aβ pathology (Cancino et al, 2008) and parkin relationship with amyloid accumulation (Perucho et al, 2010) were previously reported in AD models, our results identified novel mechanisms involving TKI-mediated autophagic clearance of intraneuronal Aβ and Tau and demonstrated the effects of brain-penetrant TKIs (Bosutinib and Nilotinib) in improving amyloid pathology and cognition. These novel findings potentially have high medical impact due to lack of effective drug treatment for AD and other neurodegenerative diseases, involving intraneuronal accumulation of proteins, including the Tauopathies and α-Synucleinopathies. Additionally, penetration of well tolerated TKIs into the brain to clear intraneuronal amyloid and reduce plaque load, contrasts with anti-Aβ vaccine therapies that may reduce extracellular plaques but fail to rescue neurons from intracellular amyloid stress, leading to progression of cell death. Furthermore, the current findings show that TKI-mediated autophagy may reduce p-Tau, indicating that autophagy may clear free unbound p-Tau, which can potentially lead to toxic intracellular inclusions, and spare Tau that may be bound to microtubule. The decrease in p-Tau at serine and threonine residues may be due to increased autophagic clearance of this protein, but lack of detection of tyrosine phosphorylated Tau suggests that tyrosine phosphorylation of Tau may occur at a later stage of Tau pathology. These data provide TKI as a therapeutic strategy to reduce p-Tau in a number of human Tauopathies.

Beclin-1 levels were reported to decrease in AD brain (Pickford et al, 2008), and autophagic defects result in amyloid accumulation due to lack of autophagosome clearance (Nixon & Yang, 2011). However, our results suggest that Beclin-1 levels are increased in AD brains, perhaps due to different stages of disease pathology and sample extraction between our studies and those reported by (Pickford et al, 2008), but this increase in Beclin-1 is not associated with interaction with parkin, whose solubility is decreased in AD (Lonskaya et al, 2012c). Our results show that blocking Beclin-1 expression or deleting parkin impairs amyloid clearance, while others showed that lentiviral Beclin-1 expression activates autophagy in AD models (Pickford et al, 2008). TKIs stimulate autophagy (Salomoni & Calabretta, 2009), and decrease the level of insoluble parkin, leading to amyloid clearance in a parkin-dependent manner. Mutations in the gene coding for the E3-ubiquitin ligase parkin (*Park2*) are associated with inherited PD (Kitada et al, 1998). Parkin solubility is affected with many non-familial PD-linked stressors, including MP*p*+, rotenone, 6-hydroxydopamine and dopamine (Wang et al, 2005), and protein aggregates alter parkin solubility (Kawahara et al, 2008). Furthermore, decreased parkin solubility is associated with alteration of its activity via increased phosphorylation by several kinase activities, including Abl (Imam et al, 2011; Ko et al, 2010). Therefore, alteration in parkin solubility suggests that parkin activity is affected in AD.

TKs, including Abl, are activated in neurodegeneration (Imam et al, 2011; Jing et al, 2009; Ko et al, 2010; Tremblay et al, 2010) and may lead to alteration of parkin function (Imam et al, 2011; Ko et al, 2010). Therefore, TK activity may be manipulated to induce functional parkin-Beclin-1 interaction to stimulate autophagic clearance in neurodegeneration. Abl encodes a protein TK that is distributed in the nucleus and the cytoplasm of proliferating cells and is involved in a wide range of functions, including control of cell cycle and apoptosis (Wang, 2000). Aβ activates Abl and induces p-Tau suggesting that Abl participates in Aβ-induced p-Tau, while Imatinib reduces AT8 and PHF1 levels of p-Tau (Cancino et al, 2011) and reverses cognitive decline (Cancino et al, 2008; Cancino et al, 2011) in AD mice. These findings are in agreement with our data showing that TKI leads to clearance of amyloid proteins and improvement of cognitive function in AD models. Accumulation of autophagosomes in neurodegeneration may be due to reduced autophagic flux (Gonzalez-Polo et al, 2005). Parkin, Aβ and p-Tau accumulate in AVs compared to lysosomes in Tg-APP and lentiviral Aβ_1−42_ expressing mice, while TKI increases protein levels in the lysosomes, indicating autophagic flux. Autophagosome accumulation could be due to lack of maturation, leading to inefficient fusion with lysosomes and decreased autophagic clearance (He & Klionsky, 2009; Iwata et al, 2005; Kovács et al, 1982). The accumulation of LC3-II in the brain of AD models suggests autophagic defects, including autophagosome accumulation, leading to decreased amyloid clearance. TKI decreases LC3-II levels and subcellular fractionation show a decrease in Aβ and p-Tau in autophagosomal fractions (AV10 and AV20) and deposition in lysosomes, indicating that TKI facilitates autophagosome clearance. Additionally, amyloid clearance in the brain, but not blood, suggest that TKI targets intracellular amyloid. The lentiviral model confirms the role of parkin in autophagic degradation of intracellular Aβ_1−42_, leading to decreased plaques, while parkin deletion leads to more plaque formation due to lack of parkin-mediated clearance of intraneuronal Aβ_1−42_. Failure of TKIs to alter the contents of AVs and deposit amyloid in the lysosome in parkin^−/−^ mice suggest that functional parkin plays an essential role in autophagosome maturation, leading to lysosomal degradation (He & Klionsky, 2009; Iwata et al, 2005; Kovács et al, 1982). Parkin modulates Beclin-1-LC3 mediated autophagy (Chen et al, 2010) and loss of parkin function (T240R) is associated with lack of autophagosome clearance in α-Synuclein gene transfer models (Lonskaya et al, 2012b). Therefore, parkin activation may lead to increased functional interaction with Beclin-1 and enhanced autophagic clearance.

Although no parkin mutations are found in AD, manipulation of parkin activity can be a disease modifying therapy that would provide an alternative approach to prevent progression from mild cognitive impairment (MCI) to AD. Nilotinib and Bosutinib are FDA approved and enter the brain at sufficient concentrations to inhibit TKs, therefore they may be re-purposed to treat MCI. TKIs are pleiotropic drugs used in late stage CML, but AD does not involve a single pathway that may be efficiently treated with a single drug, therefore a reduction of drug dose may prevent the pleiotropic effects of TKIs. Progression from MCI to AD is a slow neurodegenerative process, and Bosutinib and Nilotinib are effective in young and aged AD mice with a lower drug dose over a longer time period, providing some proof of concept that lower dose of TKIs may be useful to halt the slow progression from MCI to AD. Although TKIs do not accumulate in the brain longer than 8 (Nilotinib) to 12 h (Bosutinib), it is possible that these drugs stimulate autophagic clearance to clear amyloid proteins that have accumulated between different treatments. Despite the promising effects of TKI in AD models, several studies previously reduced amyloid plaques in transgenic mice, but failed to halt AD progression in humans. Therefore, phase II clinical trials are needed to demonstrate the efficacy of TKI on human pathology and dementia. This is a novel mechanism involving TKI enhancement of autophagic degradation of amyloid proteins in AD mice. This approach suggests that autophagic degradation of intracellular Aβ and p-Tau can reduce extracellular plaques, leading to cognitive improvement.

## MATERIALS AND METHODS

### Human postmortem brain tissues

Human postmortem samples were obtained from John's Hopkins University brain bank. Patients' description and sample preparation are summarized in (Lonskaya et al, 2012c). Data were analysed as mean ± SEM, using two-tailed *t*-test.

### Stereotaxic injection

Lentiviral constructs encoding LacZ, or Aβ_1–42_ (Rebeck et al, 2010) were stereotaxically injected 1 × 10^6^ m.o.i bilaterally into the CA1 hippocampus of 1 year old C57BL/6 or parkin^−/−^. A Total of 6 μL lentiviral stocks were delivered at a rate of 0.2 μL/min and. All procedures were approved by the Georgetown University Animal Care and Use Committee (GUACUC).

### TKI treatment

TKIs were dissolved in DMSO and a total volume of 30μL were IP injected once a day for 3 weeks. Half the animals received DMSO and the other half received TKI in DMSO.

### Statistical analysis

All statistical analysis was performed using a GraphPad Prism, version 5.0 (GraphPad software, Inc, San Diego, CA). The number (*N*) indicates the number of independent experiments (cell culture) or number of individual animals. Asterisks designate significantly different to DMSO or as indicated, all data are presented with Mean ± SEM, with actual *p*-values obtained using ANOVA with Neumann Keuls multiple comparison.

### Western blot analysis

Brain tissues were homogenized in 1× STEN buffer (Lonskaya et al, 2012c), centrifuged at 10,000 × *g* for 20 min at 4°C, and the supernatants containing the soluble fraction of proteins were collected. The pellet was re-suspended in either 4 M urea or 30% formic acid and adjusted to pH 7 with 1 N NaOH and centrifuged at 10,000 × *g* for 20 min at 4°C, and the supernatant containing the insoluble fraction was collected. Total parkin was immunoprobed (1:1000) with PRK8 antibody as indicated (Burns et al, 2009). Rabbit polyclonal antibodies anti-Beclin-1 (1:1000) were used (Cell Signaling, Inc). A rabbit polyclonal (Pierce) anti-LC3 (1:1000) and rabbit polyclonal (Thermo Scientific) anti-actin (1:1000) were used. Rabbit polyclonal (1:1000) tubulin (Thermo Scientific) were used. Map 2 was probed (1:1000) mouse monoclonal antibody (Pierce). Lysosomal fractions were probed with (1:1000) rabbit polyclonal LAMP2a antibodies (Abcam), BACE-1 was probed (1:1000) with rabbit monoclonal antibody (Thermo Scientific), ADAM-10 was probed with (1:1000) rabbit polyclonal antibodies (Abcam), and presenilin-1 was probed with (1:1000) rabbit polyclonal (Cell Signaling). All WBs were quantified and expressed as % control.

### Immunohistochemistry

Immunohistochemistry was performed on 20 micron-thick 4% paraformaldehyde (PFA) fixed cortical brain sections. Aβ_1–42_ was probed (1:200) with rabbit polyclonal specific anti-Aβ_1–42_ antibody (Zymed) that recognizes a.a. 1–42, and (1:200) mouse monoclonal antibody (4G8) that recognizes a.a. 17–24 (Covance) and counterstained with DAPI. Parkin was immunoprobed (1:200) with mouse anti-parkin (PRK8) antibody that recognizes a.a. 399–465 (Signet Labs, Dedham, MA) and rabbit polyclonal (1:200) anti-parkin (AB5112) antibody that recognizes a.a. 305–622 (Millipore) and counterstained with DAPI. Mouse monoclonal (6E10) antibody (1:100) with DAB were used (Covance) and thioflavin-S was performed according to manufacturer's instructions (Sigma).

### Stereological methods

Stereological methods were applied by a blinded investigator using unbiased stereology analysis (Stereologer, Systems Planning and Analysis, Chester, MD) as described in (Lonskaya et al, 2012c; Rebeck et al, 2010).

### Proximity ligation assay (PLA)

Primary 1:100 mouse anti-parkin (PRK8, above) and rabbit 1:100 anti-Beclin-1 (above) antibodies were applied to 20 μm thick sections of mouse brain or de-parrafanized PPE human brains overnight at 4°C. Duolink In Situ Red Starter Kit (Cat#92101-KI01) containing species-specific secondary antibodies or PLA probes, each with a unique short DNA strand attached to it (Axxora, LLC, Farmingdale, NW) was used as described in manufacturer's protocol. When the PLA probes are in close proximity, the DNA strands interact through a subsequent addition of two other circle-forming DNA oligonucleotides. After joining of the two added oligonucleotides by enzymatic ligation, they are amplified via rolling circle amplification using a polymerase to highlight the interaction. Fluorescence in each single-molecule amplification product is easily visible as a distinct bright spot when viewed with a fluorescence microscope.

### Aβ and p-Tau ELISA

Aβ and p-Tau enzyme-linked immunosorbent assay (ELISA) using specific p-Tau, Aβ_1–40_ and Aβ_1–42_ ELISA and caspase-3 activity were performed according to manufacturer's protocol as described in (Lonskaya et al, 2012c; Rebeck et al, 2010).

### Subcellular fractionation to isolate autophagic vacuoles

0.5 g of Frozen human or animal brains were homogenized at low speed (Cole-Palmer homogenizer, LabGen 7, 115 Vac) in 1×STEN buffer and centrifuged at 1000 × *g* for 10 min to isolate the supernatant from the pellet. The pellet was re-suspended in 1×STEN buffer and centrifuged once to increase the recovery of lysosomes. The pooled supernatants were then centrifuged at 100,000 rpm for 1 h at 4°C to extract the pellet containing AVs and lysosomes. The pellet was then re-suspended in 10 mL (0.33 g/mL) 50% Metrizamide and 10 mL in cellulose nitrate tubes. A discontinuous Metrizamide gradient was constructed in layers from bottom to top as follows: 6 mL of pellet suspension, 10 mL of 26%; 5 mL of 24%; 5 mL of 20% and 5 mL of 10% Metrizamide (Marzella et al, 1982). After centrifugation at 10,000 rpm for 1 h at 4°C, the fraction floating on the 10% layer (Lysosome) and the fractions banding at the 24%/20% (AV 20) and the 20%/10% (AV10) Metrizamide inter-phases were collected by a syringe and examined.

### Cell culture and transfection

Human neuroblastoma M17 or rat B35 cells were grown in 24 well dishes (Falcon) as previously described (Lonskaya et al, 2012c; Rebeck et al, 2010). Transient transfection was performed with 3 µg Aβ_1–42_ cDNA, or 3 µg LacZ cDNA for 24 h. Cells were treated with 10 μM Nilotinib for 24 h. Cells were harvested 48 h after transfection. Cells were harvested one time with STEN buffer and centrifuged at 10,000 × *g* for 20 min at 4°C, and the supernatant was collected.

### Parkin ELISA

Parkin ELISA was performed on brain soluble brain lysates (in STEN buffer) or insoluble brain lysates (4 M urea) using mouse specific parkin kit (MYBioSource) in 50 µL (1 µg/µL) of brain lysates detected with 50 µL primary antibody (3 h) and 100 µL anti-rabbit antibody (30 min) at RT. Extracts were incubated with stabilized Chromogen for 30 min at RT and solution was stopped and read at 450 nm, according to manufacturer's protocol.

### Morris water maze

All animals were pre-trained (trials) to swin for 90 s in a water maze containing a platform submerged in water (invisible) for 4 consecutive days once a day. The pretraining trials ‘teach’ the swimming animals that to ‘escape’, they must find the hidden platform, and stay on it. The water maze ‘test’ was performed on day 5 (D'Hooge and De Deyn, 2001), when the platform was removed and mice have to swin and find it, thus assessing acquisition and retention. All parameters, including distance travelled to reach platform, speed to get to the platform, latency or time spent on platform and platform entry were digitally recorded on a computer and analysed by a blind investigator.

### Quantification of plaque load

Quantification of plaque load or counting plaque number was performed by a blind investigator using ImageJ by drawing a line around individual plaques within 1 mm^2^ radius of 6 randomly selected hippocampal and cortical regions in 6E10 stained slides. The number of plaques was averaged per mm^2^ and compared between treatment conditions.

### Immunoprecipitation

Mouse brains or human post-mortem tissues were homogenized in 1XSTEN buffer and the soluble fraction was isolated as indicated above. The lysates were pre-cleaned with immobilized recombinant protein G agarose (Pierce #20365), and centrifuged at 2500 × *g* for 3 min at 4°C. The supernatant was recovered, and quantified by protein assay and a total of 100 mg protein was incubated for 1 h at 4°C with primary 1:100 mouse anti-parkin (PRK8, above) and rabbit 1:100 anti-Beclin-1 (above) antibodies in the presence of sepharose G and an IgG control with primary antibodies. Parkin^−/−^ mouse brain lysates were also used to determine IP specificity. The immunoprecipiates were collected by centrifugation at 2500 × *g* for 3 min at 4°C, washed 5× in PBS, with spins of 3 min, 2500 × *g* using detergent-free buffer for the last washing step and the proteins were eluted according to Pierce instructions (Pierce #20365). After IP, the samples were size-fractionated on 4–12% SDS-NuPAGE and transferred onto 20 μm nitrocellulose membranes. The primary antibodies used for WB analysis of the parkin and Beclin-1 were the same as those used for IP. WB detection of the parkin and Beclin-1 was then performed using either HRP conjugated secondary antibodies.

Alzheimer's disease (AD) is an aging disorder that leads to memory loss. AD is characterized by intraneuronal tangles containing hyper-phosphorylated Tau (p-Tau) and extracellular β-amyloid (Aβ) plaques, derived from amyloid precursor protein (APP) cleavage and accumulation of Aβ. Abl is found within neuritic plaques and neurofibrillary tangles (NFTs) and activated (via phosphorylation) in AD post-mortem brains. Src tyrosine kinase (TK) is also recognized in AD pathology via interaction with Tau. Abl inhibition prevents Aβ_1–42_ fibrils and hydrogen peroxide (H_2_O_2_)-induced cell death, and hippocampal injection of Aβ fibrils leads to an increase of Abl levels. These data led to the hypothesis that tyrosine kinase inhibitors (TKIs) will activate parkin and facilitate autophagic amyloid clearance, thus preventing cognitive decline in AD models. We used several TKIs, including Bosutinib and Nilotinib, which penetrate the brain and induce autophagy in AD models and evaluated the effects of TKIs on parkin-mediated autophagic amyloid clearance in AD models.

RESULTS:

We identified two FDA-approved drugs, Bosutinib and Nilotinib, as potential therapies for AD. Our results indicate that decreased parkin solubility decreases functional interaction with a key autophagy molecule, Beclin-1, while TKIs reverse parkin-Beclin-1 interaction in AD models, leading to autophagic clearance of amyloid proteins. TKI-induced decrease of intraneuronal Aβ_1–42_ led to plaque disappearance while lentiviral production of intraneuronal Aβ_1–42_ resulted in plaque formation. Importantly, parkin is required for autophagosome maturation and autophagic clearance and TKIs enhance amyloid clearance and cognitive performance in a parkin-dependent manner.

IMPACT:

Our studies show that TKI could be a therapeutic strategy, via degradation of amyloid proteins, in neurodegenerative diseases, including AD. We identified two FDA-approved drugs as potential therapies for AD. TKIs are well tolerated in human leukaemia patients and could be used with a smaller dose over a longer period of time to prevent progression from mild cognitive impairment (MCI) to AD.

## Author contributions

IL performed prepared lentivirus, IHC and Morris Water Maze, MLH performed ELISA, parkin activity assays and Western blots, NMD and AF performed WB. CE-HM injected the animals, oversaw the studies and wrote the manuscript.

## Supporting Information

As a service to our authors and readers, this journal provides supporting information supplied by the authors. Such materials are peer reviewed and may be re-organized for online delivery, but are not copy-edited or typeset. Technical support issues arising from supporting information (other than missing files) should be addressed to the authors.
