# Supplementary material for: Tyrosine kinase inhibition increases functional parkin-Beclin-1 interaction and enhances amyloid clearance and cognitive performance
Source: EMBO Mol Med. 2013 Jul 4;5(8):1247–62. doi: 10.1002/emmm.201302771 (PMC3944464; doi:10.1002/emmm.201302771)
Supplement: Supplementary file 1 [file emmm0005-1247-SD1.pdf]

Tyrosine kinase inhibition increases functional parkin-Beclin-1 interaction and enhances amyloid clearance and cognitive performance

Irina Lonskaya, Michaeline L. Hebron, Nicole M. Desforges, Alexander Franjie, Charbel E-H Moussa

Corresponding author: Charbel Moussa, Georgetown University

Review timeline:

Submission date:	19 March 2013
Editorial Decision:	16 April 2013
Revision received:	26 April 2013
Editorial Decision:	10 May 2013
Revision received:	10 May 2013
Editorial Decision:	15 May 2013
Revision received:	15 May 2013
Editorial Decision:	21 May 2013
Revision received:	21 May 2013
Accepted:	22 May 2013

Transaction Report:

(Note: With the exception of the correction of typographical or spelling errors that could be a source of ambiguity, letters and reports are not edited. The original formatting of letters and referee reports may not be reflected in this compilation.)

Editor: Roberto Buccione

1st Editorial Decision 16 April 2013

Thank you for the submission of your manuscript to EMBO Molecular Medicine. We have now heard back from the three Reviewers whom we asked to evaluate your manuscript.
You will see that two Reviewers are generally supportive of you work while one is quite negative. All considered, the issues raised prevent us from considering publication at this time. I will not dwell into much detail, as the evaluations are detailed and self-explanatory. I would like, however, to highlight a few main points.

Reviewer 1 notes a number of experimental issues that require your action. For instance s/he would like you to quantify pAbl as a proportion of total Abl and to confirm the proposed Beclin-1/Parkin interaction by co-immunoprecipitation. Reviewer 1 is also concerned that important controls are missing in your experiments. This Reviewer also lists other critical issues that require your intervention.

Reviewer 2 would like you to tone down some over-emphasised conclusions considered that the predictive power of drug studies in mouse models for AD has been far from high.

Reviewer 3's main concern is that your manuscript suffers from lack of novelty. S/he does recognise however, the potentially high medical impact of your findings. Reviewer 3 also notes other items of concern including
(similarly to Reviewer 1) the need for further confirmation of the Beclin-1/Parkin interaction, to verify the possible effect of the inhibitors on APP processing and raises doubts on the significance of the cognitive tests. I am prepared to override the novelty issue, provided all Reviewer 3's other concerns are fully addressed and that you carefully clarify better and where applicable the novelty of current results related to the previously published data from your group and others.

Considered all the above, while publication of the paper cannot be considered at this stage, we would be prepared to consider a substantially revised submission, with the understanding that the Reviewers' concerns must be fully addressed, (with the exception noted above) with additional experimental data where appropriate and that acceptance of the manuscript will entail a second round of review.

Please note that it is EMBO Molecular Medicine policy to allow a single round of revision only and that, therefore, acceptance or rejection of the manuscript will depend on the completeness of your responses included in the next, final version of the manuscript.

As you know, EMBO Molecular Medicine has a "scooping protection" policy, whereby similar findings that are published by others during review or revision are not a criterion for rejection. However, I do ask you to get in touch with us after three months if you have not completed your revision, to update us on the status. Please also contact us as soon as possible if similar work is published elsewhere.

I look forward to seeing a revised form of your manuscript as soon as possible.

***** Reviewer's comments *****

Referee #1 (General Remarks):

This is a very interesting study, which extends previous studies by the author to show a critical pathogenic role of Beclin-1-Parkin interactions in mouse models of AD. This manuscript shows that FDA-approved tyrosine kinase inhibitors stimulate autophagy to decrease amounts of insoluble parkin, the increased soluble parkin acting to strengthen parkin-beclin-1 interactions, and increase Abeta and phospho-tau clearance in a parkin-dependent manner. Increased cognition in mice expressing human Abeta was also observed suggesting that this strategy may have therapeutic benefit in the treatment of AD.

Robust pharmacological treatments for AD remain a huge unmet need, and this paper provides strong support for further investigations into tyrosine kinase inhibitors as a potential treatment option. Moreover, the authors show convincing evidence that parkin-Beclin-1 interactions are intrinsically involved in the pathological accumulation of Abeta and phospho-tau in models of AD.

I have no strong criticisms of this work, only a few, relatively minor, comments:

1. In Figure 1A, the authors show concentration of TKIs in the brain of WT mice over time. This data should be extended to show amounts of TKI, or at least bosutinib, for 24 and 48 hours after injection. At present it is not clear if any TKIs remain in the brain 24 hours post-injection and if there are any cumulative effects over time. This is particularly relevant for the experiments described in Figure 3 where a 2-day dosing regime was used.
2. pT412 Abl should be quantified as a proportion of total Abl, not as a proportion of the loading control (Fig. 1B and also Fig 4E). This will indicate whether or not differences in the activity of Abl are real or an artefact of the decreased total abl amounts observed.

3. Duo-link experiments showing Beclin-1 and Parkin interaction in mouse and human brain should be confirmed by co-innumoprecipitation if possible, to allow determination of whether or not this is a direct interaction.
4. In Figure 1P, the amount of Beclin-1 in Bosunitib and vehicle-treated APP Tg mice is variable. These findings should be quantified with respect to amounts of tubulin or another protein loading control.
5. It is important to show parkin, beclin-1, Abl, pAbl and LC3 protein amounts following TKI treatment of WT, APP Tg, LV-Abeta and parkin deficient mice to allow proper interpretation of the data (similar to that shown in Fig 1B for APP Tg treated mice). In addition, protein amounts of lysosome markers (e.g. LAMP1 or other appropriate markers) should be shown to ensure that the results shown in Fig. 5B have not arisen due to experimental artefacts. Similarly, if possible, specific markers of early forming autophagic vacuoles (AV10) and auophagosomes (AV20) should be shown to complement/strengthen Fig 5.
6. The results shown in Fig 3F are quite remarkable. Can the authors please speculate further on the mechanism by which TKIs reduce phosphorylation of tau at Ser/Thr residues. Have they examined tau phosphorylation at Tyr sites? since ser/thr phosphorylation has previously been shown to affect tau interaction with some src kinases (Reynolds et al., 2008). Antibodies against Tyr18 (Fyn) and Tyr394 (Abl) have previously been described by others.

Typos/minor remarks

1. Are the results in Fig. 4C and 4D also from WT mice? This information is missing from the text/fig legend.
2. Fig 4E is mis-labelled as Fig 4C in the figure legend.
3. Can the lines indicating significance in Figures 4, 5 and 6 please be re-drawn. It is very difficult to interpret the columns indicated at present.

Referee #2 (Comments on Novelty/Model System):

This manuscript describes inhibition of c-abl (and perhaps other tyrosine kinases) as a novel approach to increasing autophagy and reducing Abeta deposition. The studies are very well done, although there are some concerns about the positive predictive value of drug studies in APP mice.

Referee #2 (General Remarks):

This is a well done study, and describes interesting results. The idea that c-abl regulates parkin and that the parkin-beclin interaction regulates autophagy seems to be well supported by the data. The biochemical analysis is comprehensive. One minor point that would assist comparison with similar studies in these mice is to present a figure showing amyloid staining as "percent area stained" - what is often called "amyloid burden". As the authors have these sections already stained with 6E10, perhaps this could be included. While the authors enthusiasm for tyrosine kinase inhibitors as therapeutics is admirable, perhaps it could be tempered with a statement about the predictive power of drug studies in APP mice. These have so far not been successful at predicting success in human AD patients.

Referee #3 (General Remarks):

This manuscript describes that tyrosine kinase inhibitors (TKI) targeting Src/Abl kinases reduce Ab

and amyloid plaque clearance and reverse cognitive deficits in AD mouse models by a parkin-dependent mechanism. The results are of potential interest because TKI, which are used in leukemia patients, could have therapeutic benefits in AD conditions. In my opinion, the major novelty of the current results is that the inhibitors Nilotinib and Bosutinib reduce Ab-related pathology and behavior in TgAPP mice by enhancing parkin-beclin interaction, which are suppose to enhance autophagic Ab clearance (this study; Khandelwal et al., 2011). However, some of these results partially overlap with previous reports. Thus, these authors and others showed previously that parkin decreases intraneuronal Ab42 (Burns et al., 2009; Rosen et al., 2010), extracellular plaques and other deficits in AD models through ubiquitination and autophagic mechanisms (Lonskaya et al., 2013). The same authors recently showed that clearance of Ab is mediated by parkin-mediated beclin-dependent autophagy (Khandelwal et al., 2011). In addition, c-Abl TKI STI571 has been shown to reduce amyloid plaques and cognitive decline in APPswe/PS1D9 transgenic mice (Cancino et al., 2008). These previous evidence partially limit the novelty of the present molecular results. Those include: changes on parkin and LC3-II in TgAPP brains (Fig. 1) already shown in AD brains (Lonskaya et al., 2013); parkin/beclin-mediated autophagic Ab clearance in 3xTg-AD mice (Khandelwal et al., 2011); reduced Ab and plaque deposition in TgAPP mice by the dual Src/Abl inhibitor bosutinib (Fig 2 and 3) already shown with an Abl inhibitor (Cancino et al., 2008); and improvement of cognitive performance in TgAPP mice by bosutinib (Fig. 6) similarly reported by Abl and Src inhibitors (Cancino et al., 2008; Dhawan and Combs, 2012). In addition, parkin suppression reduces Ab peptides, amyloid plaques and tau phosphorylation and memory deficits in APP transgenic mice by an autophagy mechanism (Perucho et al., 2010). These evidences limit the impact of the results, which are not sufficiently novel for publication in EMBO Mol Med.

Other comments:

- The effect of TKI on parkin-beclin interaction could be further confirmed by biochemical analyses (CoIPs..)
- Number of amyloid plaques in vehicle and bosutinib-treated mice should be quantified (Fig 2 and 4G-J)
-The authors have not evaluated a possible effect of TKI on alpha,beta and gamma-secretase processing of APP which could lead to reduced Ab levels/deposition.
- The authors claim some significant differences on quadrant occupancy and number of platform entries (SE quadrant) in bos and vehicle APPtg mice (Figs 6D,E). However, the mean (sem) values shown in the graphs are so high to believe that those differences are significant.

1st Revision - authors' response 26 April 2013

Response to reviewers.

On behalf of all authors, I would like to thank you and the reviewers for your assistance in the review of this manuscript. I attached the revised manuscript taking into account all 100% of 3 reviewers' comments. I believe that the comments were highly reasonable and encouraging and all the additional experiments that were requested surely strengthened the message of this manuscript. I particularly want to express my gratitude to you for assisting us to address the novelty of our findings.

Below is a detailed point-by-point response how we addressed all the comments. We also highlighted these changes in the text.

Reviewer 1 notes a number of experimental issues that require your action. For instance s/he would like you to quantify pAbl as a proportion of total Abl and to confirm the proposed Beclin-1/Parkin interaction by co-immunoprecipitation. Reviewer 1 is also concerned that important controls are missing in your experiments. This Reviewer also lists other critical issues that require your intervention. We addressed all concerns. We agree with the reviewer and added new experimental data, many of which were conducted/obtianed before the submission of the paper. The criticisms were highly constructive.
Reviewer 2 would like you to tone down some over-emphasised conclusions considered that the predictive power of drug studies in mouse models for AD has been far from high. We agree with the reviewer and we toned down the language, particularly in our conclusion statement, which included a mention of the power of AD drugs in mice. The changes are highlighted in red in the discussion.

Reviewer 3's main concern is that your manuscript suffers from lack of novelty. S/he does recognise however, the potentially high medical impact of your findings. Reviewer 3 also notes other items of concern including (similarly to Reviewer 1) the need for further confirmation of the Beclin-1/Parkin interaction, to verify the possible effect of the inhibitors on APP processing and raises doubts on the significance of the cognitive tests. I am prepared to override the novelty issue, provided all Reviewer 3's other concerns are fully addressed and that you carefully clarify better and where applicable the novelty of current results related to the previously published data from your group and others.

We thank you for providing us with the opportunity to explain the significant novelty of our findings. We added the new experiments that this reviewer requested and included detailed comparison how our results differ from exciting reports published by our lab and others as the reviewer listed them (detailed in the first paragraph of the discussion). We thank this reviewer also for the constructive comments and for recognizing the translational potentials of these studies. The changes are highlighted in the text and explained below.

Referee #1 (General Remarks):

This is a very interesting study, which extends previous studies by the author to show a critical pathogenic role of Beclin-1-Parkin interactions in mouse models of AD. This manuscript shows that FDA-approved tyrosine kinase inhibitors stimulate autophagy to decrease amounts of insoluble parkin, the increased soluble parkin acting to strengthen parkin-beclin-1 interactions, and increase Abeta and

phospho-tau clearance in a parkin-dependent manner. Increased cognition in mice expressing human Abeta was also observed suggesting that this strategy may have therapeutic benefit in the treatment of AD.

Robust pharmacological treatments for AD remain a huge unmet need, and this paper provides strong support for further investigations into tyrosine kinase inhibitors as a potential treatment option. Moreover, the authors show convincing evidence that parkin-Beclin-1 interactions are intrinsically involved in the pathological accumulation of Abeta and phospho-tau in models of AD.
We thank the reviewer for his comments and the constructive input into this paper.
I have no strong criticisms of this work, only a few, relatively minor, comments:

1. In Figure 1A, the authors show concentration of TKIs in the brain of WT mice over time. This data should be extended to show amounts of TKI, or at least bosutinib, for 24 and 48 hours after injection.

We have analyzed the drugs up to 24 hr and found no traces in the brain after 18 hr. The data were added in Fig. 1A.

At present it is not clear if any TKIs remain in the brain 24 hours post-injection and if there are any cumulative effects over time. This is particularly relevant for the experiments described in Figure 3 where a 2-day dosing regime was used.

We added a statement in the discussion explaining potential effects of the drug without accumulation as follow:

Although TKIs do not accumulate in the brain longer than 8 (Nilotinib) to 12hr (Bosutinib), it is possible that these drugs stimulate autophagic clearance (turn-on) as long as they are available in the brain (turn-off), while the next dose clears amyloid proteins that have accumulated between different treatments.

2. pT412 Abl should be quantified as a proportion of total Abl, not as a proportion of the loading control (Fig. 1B and also Fig 4E). This will indicate whether or not differences in the activity of Abl are real or an artefact of the decreased total abl amounts observed.

We did that and included other conditions as this reviewer suggested (below) in Fig. 4F, thanks
3. Duo-link experiments showing Beclin-1 and Parkin interaction in mouse and human brain should be confirmed by co-innumoprecipitation if possible, to allow determination of whether or not this is a direct interaction.

This was done and data were added in Fig. 1D &E. Thanks

4. In Figure 1P, the amount of Beclin-1 in Bosunitib and vehicle-treated APP Tg mice is variable. These findings should be quantified with respect to amounts of tubulin or another protein loading control.

We deleted Fig. 1P because the data were included in Fig. 1D and performed densitometry as the reviewer requested.
5. It is important to show parkin, beclin-1, Abl, pAbl and LC3 protein amounts following TKI treatment of WT, APP Tg, LV-Abeta and parkin deficient mice to allow proper interpretation of the data (similar to that shown in Fig 1B for APP Tg treated mice).

We added these conditions in Fig. 4F (control and Abeta in WT and parkin-/-). Tg-APP are shown in figure 1. Thanks

In addition, protein amounts of lysosome markers (e.g. LAMP1 or other appropriate markers) should be shown to ensure that the results shown in Fig. 5B have not arisen due to experimental artefacts. Similarly, if possible, specific markers of early forming autophagic vacuoles (AV10) and auophagosomes (AV20) should be shown to complement/strengthen Fig 5.

We added these data as an insert in Fig. 5A.

6. The results shown in Fig 3F are quite remarkable. Can the authors please speculate further on the mechanism by which TKIs reduce phosphorylation of tau at Ser/Thr residues. Have they examined tau phosphorylation at Tyr sites? since ser/thr phosphorylation has previously been shown to affect tau interaction with some src kinases (Reynolds et al., 2008). Antibodies against Tyr18 (Fyn) and Tyr394 (Abl) have previously been described by others.

We share the same impression with the reviewer and we did actually examine Tau phosphorylation at Tyr sites. Unfortunately we did not get any data but we discussed this in the results and discussion section. We did not mention this in the previous submission because we are performing experiments in older Tg-APP animals to see whether we can detect Tyr-phosphorylated Tau at later stages of Tau pathology.

Results:

No phospho-tyrosine Tau was detected with the commercially available antibody (4G10, Millipore) and immunoprecipitation of total Tau (Tau-5 antibody) and probing with total phospho-tyrosine did not show any difference between control and Tg-APP mice (data not shown), suggesting that Tau phosphorylation at Ser and Thr may affect phosphorylation at tyrosine residues at later stages of Tau pathology.

Discussion

The decrease in p-Tau at serine and threonine residues may be due to increased autophagic clearance of this protein, but lack of detection of tyrosine phosphorylated Tau suggests that tyrosine phosphorylation of Tau may occur at a later stage of Tau pathology.

Typos/minor remarks

1. Are the results in Fig. 4C and 4D also from WT mice? This information is missing from the text/fig legend. We added the info to the figure legend. Thanks
2. Fig 4E is mis-labelled as Fig 4C in the figure legend. Corrected
3. Can the lines indicating significance in Figures 4, 5 and 6 please be re-drawn. It is very difficult to interpret the columns indicated at present. We re-drew the lines, sorry about the confusion.

Referee #2 (Comments on Novelty/Model System):

This manuscript describes inhibition of c-abl (and perhaps other tyrosine kinases) as a novel approach to increasing autophagy and reducing Abeta deposition. The studies are very well done, although there are some concerns about the positive predictive value of drug studies in APP mice.

We thank the reviewer for the supportive and encouraging comments. All concerns were addressed below.

Referee #2 (General Remarks):

This is a well done study, and describes interesting results. The idea that c-abl regulates parkin and that the parkin-beclin interaction regulates autophagy seems to be well supported by the data. The

biochemical analysis is comprehensive. One minor point that would assist comparison with similar studies in these mice is to present a figure showing amyloid staining as "percent area stained" - what is often called "amyloid burden". As the authors have these sections already stained with 6E10, perhaps this could be included.

We added the data in Fig. 2S. These analyses were performed previously and we thank the reviewer for requesting the data.

While the authors enthusiasm for tyrosine kinase inhibitors as therapeutics is admirable, perhaps it could be tempered with a statement about the predictive power of drug studies in APP mice. These have so far not been successful at predicting success in human AD patients.

We agree with the reviewer. We toned down over-emphasized statements in the manuscript and added a new statement in the discussion highlighting the limitations of AD drug therapies as follow: Progression from MCI to AD is a slow neurodegenerative process, and Bosutinib and Nilotinib are effective in young and aged AD mice with a lower drug dose over a longer time period, providing some proof of concept that lower dose of TKIs may be useful to halt the slow progression from MCI to AD. Although TKIs do not accumulate in the brain longer than 8 (Nilotinib) to 12hr (Bosutinib), it is possible that these drugs stimulate autophagic clearance (turn-on) as long as they are available in the brain (turn-off), while the next dose clears amyloid proteins that have accumulated between different treatments. However, it should be mentioned that several studies successfully reduced the level of amyloid proteins in transgenic AD models, and failed promising effects in human patients, suggesting that phase II clinical trials are needed to demonstrate the efficacy of TKI on human pathology and dementia. This is a novel mechanism involving TKI-induced enhancement of autophagic degradation of amyloid proteins in AD mice.

Referee #3 (General Remarks):

This manuscript describes that tyrosine kinase inhibitors (TKI) targeting Src/Abl kinases reduce Ab and amyloid plaque clearance and reverse cognitive deficits in AD mouse models by a parkin-dependent mechanism. The results are of potential interest because TKI, which are used in leukemia patients, could have therapeutic benefits in AD conditions. In my opinion, the major novelty of the current results is that the inhibitors Nilotinib and Bosutinib reduce Ab-related pathology and behavior in TgAPP mice by enhancing parkin-beclin interaction, which are suppose to enhance autophagic Ab clearance (this study; Khandelwal et al., 2011). However, some of these results partially overlap with previous reports. Thus, these authors and others showed previously that parkin decreases intraneuronal Ab42 (Burns et al., 2009; Rosen et al., 2010), extracellular plaques and other deficits in AD models through ubiquitination and autophagic mechanisms (Lonskaya et al., 2013). The same authors recently showed that clearance of Ab is mediated by parkin-mediated beclin-dependent autophagy (Khandelwal et al., 2011). In addition, c-Abl TKI STI571 has been shown to reduce amyloid plaques and cognitive decline in APPswe/PS1D9 transgenic mice (Cancino et al., 2008). These previous evidence partially limit the novelty of the present molecular results. Those include: changes on parkin and LC3-II in TgAPP brains (Fig. 1) already shown in AD brains (Lonskaya et al., 2013); parkin/beclin-mediated autophagic Ab clearance in 3xTg-AD mice (Khandelwal et al., 2011); reduced Ab and plaque deposition in TgAPP mice by the dual Src/Abl inhibitor bosutinib (Fig 2 and 3) already shown with an Abl inhibitor (Cancino et al., 2008); and improvement of cognitive performance in TgAPP mice by bosutinib (Fig. 6) similarly reported by Abl and Src inhibitors (Cancino et al., 2008; Dhawan and Combs, 2012). In addition, parkin suppression reduces Ab peptides, amyloid plaques and tau phosphorylation and memory deficits in APP transgenic mice by an autophagy mechanism

(Perucho et al., 2010). These evidences limit the impact of the results, which are not sufficiently novel for publication in EMBO Mol Med.

We thank the reviewer for this comprehensive summary, which aided us in highlighting the significant novelty of our novel data compared to published literature, including our own group. We provided a detailed response in the first paragraph of the discussion as follow:

These studies evaluated the effects of TKI on parkin-Beclin-1 interaction and modulation of autophagic amyloid clearance in AD models. Here we show novel mechanisms of parkin-Beclin-1 interaction, which is dependent on parkin stability as increased levels of insoluble parkin in AD and Tg-APP mice lead to loss of parkin-Beclin-1 interaction, perhaps impairing autophagic amyloid clearance. These data, along with the identification of brain penetrant FDA-approved drugs, are novel mechanistic and translational findings. These results demonstrate the impact of decreased parkin solubility (Lonskaya et al, 2012b; Lonskaya et al, 2013), which co-localizes with intraneuronal $A\beta_{1-42}$ in post-mortem AD brains (Lonskaya et al, 2012c), suggesting failure to facilitate amyloid clearance. We previously reported that exogenous parkin mediates autophagic clearance (Burns et al, 2009; Khandelwal et al, 2011; Lonskaya et al, 2012b; Lonskaya et al, 2013; Rebeck et al, 2010) and here we delineate the mechanisms related to parkin function via interaction with Beclin-1 to facilitate autophagosome maturation (Lonskaya et al, 2012b; Lonskaya et al, 2013), suggesting that parkin stability affects its protein clearance ability. Although the effects of osmotic pump delivery of TKIs on microgliosis (Dhawan & Combs, 2012), Aβ pathology (Cancino et al, 2008) and parkin relationship with amyloid accumulation (Perucho et al, 2010) were previously reported in AD models, our results identified novel mechanisms involving TKI-induced autophagic clearance of intraneuronal Aβ and Tau and demonstrated the effects of brain-penetrant TKIs (Bosutinib and Nilotinib) in improving amyloid pathology and cognition. These novel findings potentially have high medical impact due to lack of effective drug treatment for AD and other neurodegenerative diseases, involving intraneuronal accumulation of proteins, including the Tauopathies and α-Synucleinopathies. Additionally, penetration of well tolerated TKIs into the brain to clear intraneuronal amyloid and reduce plaque load, contrasts with anti-Aβ vaccine therapies that may reduce extracellular plaques but fail to rescue neurons from intracellular amyloid stress, leading to progression of cell death.

Other comments:

- The effect of TKI on parkin-beclin interaction could be further confirmed by biochemical analyses (CoIPs..)

We added this experiments in Fig. 1D and F. Thanks

- Number of amyloid plaques in vehicle and bosutinib-treated mice should be quantified (Fig 2 and 4G-J) . These data were added in Fig. 2S.

-The authors have not evaluated a possible effect of TKI on alpha,beta and gamma-secretase processing of APP which could lead to reduced Ab levels/deposition. These data were added in Fig.2T. and we added a statement in the results clarifying these resutls.

- The authors claim some significant differences on quadrant occupancy and number of platform entries (SE quadrant) in bos and vehicle APPtg mice (Figs 6D,E). However, the mean (sem) values shown in the graphs are so high to believe that those differences are significant.

We apologize for the misleading presentation of the data in Fig 6D&E. The data were plotted with Standard Deviations and not SEM and the Y-axis in Fig. 6D started with 50%, which showed an exaggerated SD. This was corrected to SEM and we revised the statistical analyses in Fig. 6D and E several times to confirm statistical significance.

Thank you.

2nd Editorial Decision 10 May 2013

Thank you for the submission of your revised manuscript to EMBO Molecular Medicine. We have now heard back from the two Reviewers whom we asked to evaluate your manuscript.

You will see that while the Reviewers are supportive of you work, a few issues remain to be acted upon (mostly relative to the new data) before we can accept your manuscript for publication.

Reviewer 1 notes that the new co-immunoprecipitation experiments showing the Beclin-1/Parkin interactions require additional controls and proper description in the Materials and Methods section. Furthermore, s/he would like the finding presented in Fig. 4F to be quantified and the implications of the relationship between the tau data shown in figures 3F and 5F properly discussed. Finally, Reviewer 1 suggests an in-depth check of your manuscript for English usage to improve readability and impact. I agree with this recommendation.

Reviewer 3, similarly to Reviewer 1 notes that the new co-immunoprecipitation experiments should be properly described. S/he also requires clarification for the indicated statement on page 7.

Please fully address the Reviewers' remaining concerns as quickly as possible and in any case within two weeks. Do consider that I will need to get back to Reviewer 1 to check your revision, while your response to Reviewer 3 can be probably assessed at the Editorial level.

I look forward to receiving your re-revised manuscript as soon as possible

***** Reviewer's comments *****

Referee #1 (General Remarks):

Lonskaya et al. present convincing and interesting data showing a beneficial impact of FDA-approved tyrosine kinase inhibitors in models of AD via increased parkin solubility, enhanced parkin-beclin-1-interactions and stimulation of autophagy.

The majority of my, and the other reviewer's, previous concerns have now been addressed. However, I have a few remaining comments:

1. The authors have now performed co-IPs to demonstrate Beclin-1/Parkin interactions. Negative (IgG) controls -primary and secondary antibody-negative lanes - should be shown since both beclin-1 and parkin run close to 50kDa. Also, the co-IP method should be described in the materials and methods section.

2. New data has been included to show the effect of bosutinib treatment on lentiviral Abeta1-42 expressing mice. The authors state that they observed a significant decrease in parkin in bosutinib-treated animals compared to DMSO controls (Fig. 4F), but this is not particularly apparent in the blots shown. Can quantification of this finding be shown in a graph or this figure be replaced with a more representative blot.

3. The implications of the relationship between the tau data shown in Figures 3F and 5F should be

discussed.

4. The manuscript would benefit from a thorough proof-read. Several sections of the results section could be written more concisely. There are a number of sections that are difficult to interpret because there is no reference to comparisons being made between groups, sentences are incomplete or the language/grammer is awkward. For example, in the discussion the sentence containing 'failed promising effects in humans' and the preceding sentences should be amended. Also in Figure 2 legend 'within various brain regions in different A-B', Figure 3 legend 'Graphs represent ELISA of human A) brain... and B) blood Abeta' should be amended to ELISA of human Abeta in A) brain and B) blood. There is a repetition of the sentence beginning 'ELISA of human soluble...' at the end of Fig 3 legend and '4-12% NuPAGE gel' information in Figure 4 legend. There are also several typos e.g. '..., which had values 41% above control levels (Figure 6E). This should be below control levels.

Referee #3 (General Remarks):

The authors have now clarified the novelty of their results in the Discussion section. The following comments as result of new data can be easily addressed:
1. New experimental procedures including parkin/beclin-1 coimmunoprecipitation, plaque load quantification...need to be included in the Methods section.
2.In page 7 (Results section) the authors indicate that unaltered levels of presenilin, BACE, ADAM-10 by TKI treatment (Fig 2T) ®suggests that TKI-induced decrease in A levels is not affected by changes in APP cleavage®. Indeed, they should indicate that TKI-induced decrease in Ab levels in unlikely to be mediated by changes on expression of APP-cleaving secretases.

2nd Revision - authors' response 10 May 2013

Thank you very much indeed for the quick turnaround of this manuscript. The reviewer's comments are legitimate and we responded to all of them. We apologize for the oversight not to include the methods. We hope that this revision will lead to the acceptance of this manuscript.

Reviewer 1 notes that the new co-immunoprecipitation experiments showing the Beclin-1/Parkin interactions require additional controls and proper description in the Materials and Methods section.

We added the requested controls, which have been done already, to figure 1. Thanks

Furthermore, s/he would like the finding presented in Fig. 4F to be quantified and the implications of the relationship between the tau data shown in figures 3F and 5F properly discussed. Finally, Reviewer 1 suggests an in-depth check of your manuscript for English usage to improve readability and impact. I agree with this recommendation.

The discussion was expanded below and you will notice that the manuscript was rigorously edited.

Reviewer 3, similarly to Reviewer 1 notes that the new co-immunoprecipitation experiments should be properly described. S/he also requires clarification for the indicated statement on page 7.

This was done, thanks

Referee #1 (General Remarks):

Lonskaya et al. present convincing and interesting data showing a beneficial impact of FDA-approved tyrosine kinase inhibitors in models of AD via increased parkin solubility, enhanced parkin-beclin-1-interactions and stimulation of autophagy.

The majority of my, and the other reviewer's, previous concerns have now been addressed. However, I have a few remaining comments:

1. The authors have now performed co-IPs to demonstrate Beclin-1/Parkin interactions. Negative (IgG) controls -primary and secondary antibody-negative lanes - should be shown since both beclin-1 and parkin run close to 50kDa. Also, the co-IP method should be described in the materials and methods section.

We apologize for not including the controls, which was an oversight due to concerns about space limitation. Please find the controls added to figure 1E. Thanks

2. New data has been included to show the effect of bosutinib treatment on lentiviral Abeta1-42 expressing mice. The authors state that they observed a significant decrease in parkin in bosutinib-treated animals compared to DMSO controls (Fig. 4F), but this is not particularly apparent in the blots shown. Can quantification of this finding be shown in a graph or this figure be replaced with a more representative blot.

The text was corrected, parkin is increased with Bosutinib and the changes are highlighted in red with numerical representation. Thanks

WB analysis of total brain lysates (Fig. 4F) showed significantly increased parkin (42%, $p<0.05$, N=7) levels relative to actin in Bosutinib treated mice compared to DMSO with and without $A\beta_{1-42}$.

3. The implications of the relationship between the tau data shown in Figures 3F and 5F should be discussed.

This was discussed in the discussion as follows:

Furthermore, the current findings show that TKI-induced autophagy may reduce p-Tau, indicating that autophagy may clear free unbound p-Tau, which can potentially lead to toxic intracellular inclusions, and spare Tau that may be bound to microtubule. The decrease in p-Tau at serine and threonine residues may be due to increased autophagic clearance of this protein, but lack of detection of tyrosine phosphorylated Tau suggests that tyrosine phosphorylation of Tau may occur at a later stage of Tau pathology. These data provide TKI as a therapeutic strategy to reduce p-Tau in a number of human Tauopathies.

4. The manuscript would benefit from a thorough proof-read. Several sections of the results section could be written more concisely. There are a number of sections that are difficult to interpret because there is no reference to comparisons being made between groups, sentences are incomplete or the language/grammer is awkward. For example, in the discussion the sentence containing 'failed promising effects in humans' and the preceding sentences should be amended. Also in Figure 2 legend 'within various brain regions in different A-B', Figure 3 legend 'Graphs represent ELISA of human A) brain... and B) blood Abeta' should be amended to ELISA of human Abeta in A) brain and B) blood. There is a repetition of the sentence beginning 'ELISA of human soluble...' at the end of Fig 3 legend and '4-12% NuPAGE gel' information in Figure 4 legend. There are also several typos e.g. '..., which had values 41% above control levels (Figure 6E). This should be below control levels.

We apologize for the sloppiness. We have read the manuscript, corrected the typos (highlighted in red) and thoroughly edited the results and discussion. Thanks

Referee #3 (General Remarks):

The authors have now clarified the novelty of their results in the Discussion section. The following comments as result of new data can be easily addressed:
1. New experimental procedures including parkin/beclin-1 coimmunoprecipitation, plaque load quantification...need to be included in the Methods section.

This was added in red at the end of the methods. Thanks

2.In page 7 (Results section) the authors indicate that unaltered levels of presenilin, BACE, ADAM-10 by TKI treatment (Fig 2T) ¨suggests that TKI-induced decrease in Aβ levels is not affected by changes in APP cleavage¨. Indeed, they should indicate that TKI-induced decrease in Ab levels in unlikely to be mediated by changes on expression of APP-cleaving secretases.

We amended the sentence as per the reviewer’s request. Thanks

3rd Editorial Decision 15 May 2013

Thank you for the submission of your revised manuscript to EMBO Molecular Medicine. We have now heard back from Reviewer 1 whom we asked to evaluate your manuscript.

Unfortunately, you will see that, in addition to pointing out a number of typos and errors that remain in the manuscript, s/he reports a potential caveat that requires your action before we can go forward with your manuscript.

Reviewer 1 notes that Figures 1B and 4F report a reduction of LC3-II following bosutinib treatment which indicates a decrease, not an induction, of autophagy. This represents a contradiction with respect to the data presented in Fig.5 and does not support the conclusion that bosutinib stimulates autophagy.

If this had been a further-reaching request from Reviewer 1, I would have waived it, but I am sure you will understand that this is not the case and that the issue requires clarification.

I look forward to seeing a revised form of your manuscript including clarification as soon as possible.

***** Reviewer's comments *****

Referee #1 (General Remarks):

Lonskaya et al. have addressed my previous comments, and provide interesting data showing a beneficial effect of TKIs in models of AD.

However, in the process of re-reviewing this manuscript, I came across what appears to be a major discrepancy in the findings that has important consequences for the conclusions of the manuscript:

In Figures 1B and 4F, data shows a reduction of LC3-II expression following bosutinib treatment. The authors conclude that this indicates that bosutinib stimulates induction of autophagy. However, decreased LC3-II represents autophagy reduction/inhibition (Klionsky et al., 2012; Autophagy 8(4), 445-554) (although it is important to note that LC3-II is also subject to lysosomal turnover). Thus, these findings are at odds with the data presented in Fig. 5, and the interpretation of many other results in the paper, and do not support the conclusion that bosutinib stimulates autophagy to promote Abeta and phospho-tau clearance in a parkin-dependent manner. It is very important that this issue is addressed.

In addition, several typos/errors still remain:

1. In the absence of graphed data, mean +/- SD should be given for all quantified data.
2. Page 4 ".....and sacrificed the animals 2-12 hours post-injection." should be amended to ".....and sacrificed the animals 2-24 hours post-injection."
3. Figure 1 F legend "reserve" should be "reverse"
4. Figure 1H. There is odd green/yellow labeling that is very apparent when this figure is printed in black and white. What is this?
5. Page 6 and Page 8. References to plaques being 'eliminated' or 'disappeared' should be amended to reflect that plaque load is reduced, but not abolished, by bosutinib. Also, the density of 4G8 staining in Fig. 2Q appears reduced compared to that in the merged image (Fig. 2R).
6. Fig. 2T: CTRL + DMSO is missing from the figure.
7. Page 7 "Presinilin-1" should be "Presenilin-1"
8. Figure 4E - correct font issues with 'insoluble' label.
9. Figure 4E - the vehicle-treated control corresponding to the sentence "Quantitative analysis using

human A 1-42 ELISA demonstrated that WT lentiviral A 1-42 expressing mice have significantly high levels of soluble (201ng/ml) and insoluble (126ng/ml) A 1-42 compared to 1-year old control (Fig. 4E, p<0.05, N=9)" on Page 8 is absent from the figure. However, this statement is somewhat redundant since you would not expect to measure human Abeta in WT mouse brain.
10. Page 8. The author state that "WB analysis of total brain lysates (Fig. 4F) showed significantly increased parkin (42%, p<0.05, N=7) levels relative to actin in Bosutinib treated mice compared to DMSO with and without A 1-42". However, no DMSO-treated mice without Abeta are shown in Fig. 4F. Similarly "....DMSO in WT and parkin-/- mice", but samples from parkin -/- mice treated with DMSO are not shown.
11. Fig. 6B legends are overlapping.

3rd Revision - authors' response 15 May 2013

Thank you for the revision of the manuscript. We profoundly thank you for giving us the opportunity to clarify such a fundamentally important issue. We feel that there is a misunderstanding about the explanation of our results and the conclusion. Fig. 1B and 4F report total LC3-II levels, which indicate autophagosome accumulation in AD mice, suggesting induction of autophagy without clearance, but bosutinib boosts autophagic clearance. Fig 5, further examined this issue by subcellular fractionation and confirmed that LC3II accumulation is due to lack of autophagosomal digestion by the lysosomes, while TKI stimulates autophagic clearance (not induction) and deposits autophagosomes in lysosomes, leading the reduction of LC3-II and amyloids. These data are all about clearance and autophagosome maturation via beclin-1-parkin interaction and they agree with each other and with previously published data. The effect on autophagic clearance rather than induction is made clear in the title and abstract and throughout the next where no mention of induction is made. However, we added another section to the discussion clarifying this issue. In addition, we corrected all typos and replaced some scans. We hope that this explanation clarifies any confusion or misunderstanding.

Below is a point-by-point explanation and the changes are highlighted in red in the text.

Referee #1 (General Remarks):

Lonskaya et al. have addressed my previous comments, and provide interesting data showing a beneficial effect of TKIs in models of AD.

However, in the process of re-reviewing this manuscript, I came across what appears to be a major discrepancy in the findings that has important consequences for the conclusions of the manuscript:

In Figures 1B and 4F, data shows a reduction of LC3-II expression following bosutinib treatment. The authors conclude that this indicates that bosutinib stimulates induction of autophagy. However, decreased LC3-II represents autophagy reduction/inhibition ((although it is important to note that LC3-II is also subject to lysosomal turnover). Thus, these findings are at odds with the data presented in Fig. 5, and the interpretation of many other results in the paper, and do not support the conclusion that bosutinib stimulates autophagy to promote Abeta and phospho-tau clearance in a parkin-dependent manner. It is very important that this issue is addressed.

We thank the reviewer for raising this point. However, we do not claim that Bosutinib induces autophagy in our animals (where autophagy is already induced and defective), but in fact clears autophagic defects, including LC3-II accumulation. Autophagy can be induced without clearance, leading to accumulation of intracellular proteins (Klionsky et al., 2012; Autophagy 8(4), 445-554). We show in this paper, and we and others showed in previous publications, that autophagy is impaired in AD models and postmortem human brains and that may result in accumulation of LC3-II as the reviewer correctly pointed out. The mouse models and the gene transfer animals used show that b-amyloid expression impairs autophagic clearance, leading to accumulation of Ab and Tau in autophagosomes (and LC3-II level is one indicator of autophagosome accumulation/formation) and decreased lysosomal clearance. When autophagosomes are digested in lysosomes, LC3-II is also

digested and disappears, so in AD models autophagy is induced and LC3-II accumulates, without effective clearance. However, Bosutinib decreases the level of LC3-II as shown in Fig. 1B and 4F, therefore facilitating clearance and this was confirmed by subcellular fractionation (Fig. 5) showing that bosutinib leads to transfer of Tau and Ab from autophagosomal fractions (AV10 and AV20) to the lysosomes, where LC3-II is digested. Even the title of the manuscript states" Tyrosine kinase inhibition increases functional parkin-Beclin-1 interaction and enhances amyloid clearance (not induction)". We conclude that Bosutinib enhances clearance via functional parkin-beclin1 interaction, which is defective in these animal models, leading to decreased clearance. Figures 1B and 4F show increased LC3-II in DMSO treated amyloid expressing animals, but Bosutinib clears LC3-II. We stressed in the discussion and throughout the paper that Bosutinib leads to parkin-beclin-1 interaction and autophagosomal maturation, i.e. termination of autophagy via lysosomal clearance. We re-read the manuscript and made sure that reference to TKI effects is made to autophagic clearance and added a sentence to clarify that in the discussion (highlighted in red). We checked the manuscript to ascertain that there is no confusion in that matter.

In addition, several typos/errors still remain:

1. In the absence of graphed data, mean +/- SD should be given for all quantified data.
This was added. Thanks

2. Page 4 ".....and sacrificed the animals 2-12 hours post-injection." should be amended to ".....and sacrificed the animals 2-24 hours post-injection." Fixed.
3. Figure 1 F legend "reserve" should be "reverse" Fixed.

4. Figure 1H. There is odd green/yellow labelling that is very apparent when this figure is printed in black and white. What is this? Printing on our printer does not give that effect, but we replaced the figure. Thanks
5. Page 6 and Page 8. References to plaques being 'eliminated' or 'disappeared' should be amended to reflect that plaque load is reduced, but not abolished, by bosutinib.

Amended

Also, the density of 4G8 staining in Fig. 2Q appears reduced compared to that in the merged image (Fig. 2R).

This contrast of the image was fixed to show similar background in 2Q and R.

6. Fig. 2T: CTRL + DMSO is missing from the figure. There is no effect with the drug on these proteins, so indicated in the text that data are not shown since proteins are even not changing in TgAPP mice treated with the drugs and compared to APP+DMSO.

7. Page 7 "Presinilin-1" should be "Presenilin-1" Done.
8. Figure 4E - correct font issues with 'insoluble' label. Fixed.
9. Figure 4E - the vehicle-treated control corresponding to the sentence "Quantitative analysis using human Aβ1-42 ELISA demonstrated that WT lentiviral Aβ1-42 expressing mice have significantly high levels of soluble (201ng/ml) and insoluble (126ng/ml) Aβ1-42 compared to 1-year old control (Fig. 4E, $p<0.05$, N=9)" on Page 8 is absent from the figure. However, this statement is somewhat redundant since you would not expect to measure human Abeta in WT mouse brain.

The figure was corrected to add the comparison and the sentence was edited to eliminate redundancy.
10. Page 8. The author state that "WB analysis of total brain lysates (Fig. 4F) showed significantly increased parkin (42%, $p<0.05$, N=7) levels relative to actin in Bosutinib treated mice compared to DMSO with and without Aβ1-42". However, no DMSO-treated mice without Abeta are shown in Fig. 4F.

The DMSO-treated mice without Abeta is lane 1, with Abeta+DMSO is lane 4. Thanks

Similarly "....DMSO in WT and parkin-/- mice", but samples from parkin -/- mice treated with DMSO are not shown. The comparison here, as the reviewer stated above is in Abeta expressing parkin-/- mice. We are looking at parkin-/- without Abeta.

11. Fig. 6B legends are overlapping. Fixed. thanks

4th Editorial Decision 21 May 2013

Thank you for the submission of your revised manuscript to EMBO Molecular Medicine. We have now received the enclosed report from the Reviewer that was asked to re-assess it. As you will see s/he is globally supportive and I am pleased to inform you that we will be able to accept your manuscript pending the following final amendments:

1) The Reviewer suggests that you amend your description of the findings in Fig. 1 as per his/her suggestion. Provided you comply with this carefully, I will be able to deal with this revision at the Editorial level.

2) As per our Author Guidelines, the description of all reported data that includes statistical testing must state the name of the statistical test used to generate error bars and P values, the number (n) of independent experiments underlying each data point (not replicate measures of one sample), and the actual P value for each test (not merely 'significant' or 'P < 0.05').

Please submit your revised manuscript within two weeks. Needless to say, the earlier you do so the sooner we will be able to check and accept your manuscript!

I look forward to reading a new revised version of your manuscript as soon as possible.

***** Reviewer's comments *****

Referee #1 (General Remarks):

It is widely considered that increased LC3-II represents an induction of autophagy. The authors interpret their data in another way, for valid reasons that they have now explained in the response to reviewers. To aid reading and understanding of their manuscript, I suggest that they clarify this point in the results section of their manuscript by modifying the language. They say that they are careful to make no reference to autophagy induction, therefore I strongly suggest that in particular they amend their description of the findings in Fig. 1 "Bosutinib significantly decreased LC3-II relative to both LC3-I (16{plus minus}2.4, mean{plus minus}sd) and MAP-2 (29{plus minus}8, mean{plus minus}sd) levels (Fig. 1B, $p<0.05$, N=9), suggesting that Abl inhibition increases parkin and induces autophagic clearance." Perhaps amending the end of the sentence to "....stimulates autophagic clearance of proteins, including LC3-II." would remove suggestions of autophagy induction and be sufficient to avoid any ambiguity about their conclusions.

All other comments have been addressed, and I am particularly glad to note that panel 4E has been replaced. Although particularly apparent when printed, the odd labeling in this panel was also very distracting on screen.

4th Revision - authors' response 21 May 2013

Thank you for your work and help with this manuscript. We changed the statement as the reviewer recommend and added a section in the methods outlining the statistical methods used and N values, while we changed p from <0.05 to actual values. I appreciate the work you invested in the manuscript and thank all reviewers who really made this work much clearer with their comments.
